# Comparison of the therapeutic effects of human umbilical cord blood-derived mesenchymal stem cells and adipose-derived stem cells on erectile dysfunction in a rat model of bilateral cavernous nerve injury

**DOI:** 10.3389/fbioe.2022.1019063

**Published:** 2022-10-07

**Authors:** Yunrong Ti, Mengbo Yang, Xinda Chen, Ming Zhang, Jingjing Xia, Xiangguo Lv, Dongdong Xiao, Jiucun Wang, Mujun Lu

**Affiliations:** ^1^ Department of Urology and Andrology, Renji Hospital, Shanghai Institute of Andrology, School of Medicine, Shanghai Jiaotong University, Shanghai, China; ^2^ Greater Bay Area Institute of Precision Medicine, School of Life Sciences, Fudan University, Guangzhou, China; ^3^ Human Phenome Institute, Fudan University, Shanghai, China

**Keywords:** cavernous nerve injury, erectile dysfunction, secretory factors, umbilical cord blood-derived mesenchymal stem cells, adipose-derived stem cells

## Abstract

**Background:** Cavernous nerve injury (CNI) is the leading cause of erectile dysfunction (ED) after radical prostatectomy and pelvic fracture. Transplantation of human adipose-derived stem cells (ASCs) has been widely used to restore erectile function in CNI-ED rats and patients. Umbilical cord blood-derived MSCs (CBMSCs) are similarly low immunogenic but much primitive compared to ASCs and more promising in large-scale commercial applications due to the extensive establishment of cord blood banks. However, whether CBMSCs and ASCs have differential therapeutic efficacy on CNI-ED and the underlying mechanisms are still not clear.

**Materials and methods:** A bilateral cavernous nerve injury (BCNI) rat model was established by crushing the bilateral cavernous nerves. After crushing, ASCs and CBMSCs were intracavernously injected immediately. Erectile function, Masson staining, and immunofluorescence analyses of penile tissues were assessed at 4 and 12 weeks. PKH-26-labeled ASCs or CBMSCs were intracavernously injected to determine the presence and differentiation of ASCs or CBMSCs in the penis 3 days after injection. *In vitro* experiments including intracellular ROS detection, mitochondrial membrane potential assay, EdU cell proliferation staining, cell apoptosis assay, and protein chip assay were conducted to explore the underlying mechanism of CBMSC treatment compared with ASC treatment.

**Results:** CBMSC injection significantly restored erectile function, rescued the loss of cavernous corporal smooth muscles, and increased the ratio of smooth muscle to collagen. PKH-26-labeled CBMSCs or ASCs did not colocalize with endothelial cells or smooth muscle cells in the corpus cavernosum. Moreover, the conditioned medium (CM) of CBMSCs could significantly inhibit the oxidative stress and elevate the mitochondria membrane potential and proliferation of Schwann cells. Better therapeutic effects were observed in the CBMSC group than the ASC group both *in vivo* and *in vitro*. In addition, the content of neurotrophic factors and matrix metalloproteinases in CBMSC-CM, especially NT4, VEGF, MMP1, and MMP3 was significantly higher than that of ASC-CM.

**Conclusion:** Intracavernous injection of CBMSCs exhibited a better erectile function restoration than that of ASCs in CNI-ED rats owing to richer secretory factors, which can promote nerve regeneration and reduce extracellular matrix deposition. CBMSC transplantation would be a promising therapeutic strategy for CNI-ED regeneration in the future.

## Introduction

The incidence of prostate cancer has risen owing to increased male life expectancy. Among a wide range of treatments for prostate cancer, surgical resection of the prostate is the most common therapeutic option. However, the incidence of erectile dysfunction caused by cavernous nerve injury (CNI-ED) after radical prostatectomy is reported as high as 70% despite the nerve-sparing techniques (Ficarra et al., 2012), and the life quality of patients is heavily affected. Following CN injury, the damaged nerve will undergo Wallerian degeneration and reactive oxygen species increase, which play an inhibitory role in CN regeneration (Zhao et al., 2016). Subsequently, NO production available to smooth muscle decreases, leading to structural changes centered on hypoxia, oxidative stress, and apoptosis of smooth muscle cells and endothelial cells, as well as the upregulation of profibrotic cytokines that result in unavoidable fibrosis of corpus cavernosum (Magheli and Burnett, 2009; Yafi et al., 2016; Wang et al., 2019). Oral phosphodiesterase 5 inhibitors stand as the representative of conventional therapies for CNI-ED. However, its role alone as a rehabilitation strategy is unclear (Hatzimouratidis et al., 2009).

Transplantation of mesenchymal stem cells (MSCs) has been prevailing in the regime of regenerative medicine, and MSCs from adipose, bone marrow, gingiva, placenta, muscle, and induced pluripotent stem cells have been used to treat CNI-ED and demonstrate efficacy (Yang et al., 2016; Sun et al., 2017; Chen et al., 2019; Gu et al., 2020; Wu et al., 2021). However, the efficacy of cell therapy varies with the cell origins (Gu et al., 2019), and the underlying mechanism remains to be investigated. Previous studies have shown that adipose-derived stem cells (ASCs) are not only capable of differentiating into one or more phenotypes to regenerate functional cells related to erection (Lin et al., 2009; Ying et al., 2019) but also able to secrete a variety of trophic factors, including vascular endothelial growth factor (VEGF), brain-derived neurotrophic factor (BDNF), insulin-like growth factor-1 (IGF-1), and basic fibroblast growth factor (bFGF) (Yang et al., 2018; Yang W. et al., 2020), to generate promising therapeutic effects for CNI-ED rats and patients (Yang et al., 2015; Haahr et al., 2016). Allogeneic ASC therapy has been gaining increased importance because of its easy access and low immunogenicity and now been widely used in CNI-ED treatment (Yang et al., 2015; Haahr et al., 2016). Autologous ASCs transplantation presents a safe option due to its low immunogenicity. However, autologous ASCs cannot be obtained without liposuction surgery which may be troublesome for underweight patients.

Umbilical cord blood, the blood tissue in the umbilical cord of a newborn, is usually discarded as medical waste and can be collected easily (Panepucci et al., 2004). Moreover, MSCs isolated from umbilical cord blood (CBMSCs) can be obtained easily and have lower immunogenicity and are more primitive than other adult MSCs (Malgieri et al., 2010; Lee et al., 2012). Compared with ASCs, CBMSCs express similar stem cell surface markers, such as CD90, CD73, and CD105, and possess comparable osteogenic and adipogenic potential but secrete more growth factors, including VEGF, FGF, and PDGF-AA (Yoo et al., 2009). In addition, the acquisition, storage, and application of CBMSCs are becoming more and more promising due to the extensive establishment of cord blood banks. CBMSCs can be isolated and stored in the cord blood banks in advance and fetched out for quick application if patients need transplantation, which assures it of the potential for large-scale commercial applications. As is with so many advantages, CBMSCs are being widely used in treating myocardial infarction (Lee et al., 2012) and cerebral palsy (Mcdonald et al., 2017), reducing the cardiotoxicity of anti-tumor drugs (Mousa et al., 2018), alleviating liver cirrhosis (Jung et al., 2010), and so on. However, the underlying therapeutic mechanisms of CBMSCs on CNI-ED have not been made clear.

In this study, we established a CNI-ED rat model to evaluate the related therapeutic efficacy of CBMSCs. Moreover, we compared the therapeutic effects between CBMSCs and the widely used ASCs. The underlying therapeutic mechanisms that CBMSCs exert were also explored, thus gaining a more comprehensive understanding of its therapeutic effects.

## Materials and methods

### Isolation of ASCs

Briefly, discarded abdominal adipose tissues from healthy female liposuction were rinsed three times with 0.25% chloromycetin and PBS. They were cut up into small fragments and then were incubated with 0.1% collagenase type IV (Sigma-Aldrich, St. Louis, MO, United States) for 60 min at 37°C with vigorous shaking. The top lipid layer was removed after centrifugation at 1,200 g for 15 min. Then, the pellet was treated with red blood cell lysis buffer for 15 min on ice to lyse RBCs. Subsequently, the remaining cells were plated in Dulbecco’s modified Eagle’s medium (DMEM, Invitrogen Corporation) supplemented with 1% streptomycin, 1% penicillin, and 10% fetal bovine serum (FBS, Invitrogen corporation) and filtrated through a 0.22-um nylon filter mesh. The isolated ASCs were resuspended and seeded in a 10-cm dish and cultured in a 5% CO_2_ incubator at 37°C. The medium was changed every 2 days, and the cells were passaged when the fusion degree reached 80%–90%.

### Isolation and identification of CBMSCs

The isolation of CBMSCs was conducted as described previously (Meng et al., 2016). After obtaining the donor’s informed consent, the cord blood was obtained by puncture from the distal placental side of the umbilical cord, and heparin sodium (Huinuo Pharmaceutical, China) was added to anticoagulate the cord blood. After that, the same volume of red blood cell lysis buffer (STEMCELL Technologies, Vancouver, Canada) was added, and the red blood cells lysed after 15 min. After mixing with Ficoll solution (density: 1.017, Sigma-Aldrich, United States), the mixture was put into a high-speed centrifuge (Beckman, United States), and the monocytes were obtained by gradient centrifugation (3500 rpm, 37°C, 30 min), and then the monocytes were seeded into the 10-cm cell culture dish with the low-glucose medium containing 10% fetal bovine serum (FBS, Invitrogen Corporation, United States). The cells were cultured at 5% CO_2_ and 37°C in the incubator and then passed on to the culture when the fusion degree of cells was close to 90%. Finally, the cultured media of P3 cells were collected and stored in a −80°C refrigerator until further testing.

Identification of immunophenotype of CBMSCs was conducted as described in a previous study (Zhang X. et al., 2011). First, P3 of CBMSCs was blocked with 3% donkey serum and then was stained with the following fluorescence-labeled mouse anti-human antibodies: CD31-PE, HLA-DR-PE, CD73-PE, and CD90-PE (BD Biosciences, San Jose, United States). Thereafter, fluorescence was detected using the CytoFLEX cytometer (Beckman Coulter, United States).

### Adipogenic and osteogenic differentiation

Adipogenic differentiation assay was performed by culturing in the adipogenic differentiation medium (STEMCELL Technologies, Canada) for 21 days and then identified by Oil Red-O staining. Osteoblastic differentiation of CBMSCs and ASCs was induced by the osteogenic differentiation medium (STEMCELL Technologies, Canada) for 14 days and then analyzed by Alizarin Red staining.

### Isolation of rat cavernous smooth muscle cells

After anesthesia, the rat was sterilized, the foreskin and dorsal blood vessels of the penis were removed, and the cavernous tissue was obtained. The cavernous tissue was washed in PBS and cut into 1–2-mm pieces. Segments were placed on 10-cm cell culture dishes (Corning, United States) with a minimal volume of DMEM, supplemented with 20% FBS, and cultured at 37°C in a humidified atmosphere of 95% air and 5% CO_2_. After the explants were attached to the substrate, more DMEM containing 10% FBS was added, and tissue segments that had detached from the dishes were removed. The cells were frozen or passaged once 80%–90% confluence was achieved.

### PKH-26 labeling

CBMSCs and ASCs at 80%–90% confluence were digested with 0.25% trypsin, washed two times with PBS, and then labeled with PKH-26 (Sigma-Aldrich, MO, United States) according to the manufacturer’s instructions. Briefly, CBMSCs and ASCs were resuspended with 1 ml dilution C solution, and then PKH-26 ethanolic dye solution was added to the cell suspension and mixed thoroughly and then incubated for 3 min at room temperature. An equal volume of FBS was used to stop the reaction. The cells were centrifuged and washed two times with the complete medium to remove the remaining dye. The cells were injected into the corpus cavernosum of BCNI rats immediately after nerve crushing and detected by immunofluorescence staining 3 days after injection.

### Animal model and group

A total of 48 eight-week-old male Sprague–Dawley rats (mean weight = 250 g) were used in the study. All animals were housed in a standard animal facility under 12-h on and off light conditions. The rats were acclimatized for at least 1 week before surgery and allowed free access to standard food and water.

Bilateral CNI was performed in 36 rats (CNI group), and the other 12 rats were subjected to only laparotomy (sham group). In the CNI group, the construction of the CNI-ED animal model was conducted as previously described (Fang et al., 2016). After anesthetizing with 2.5% isoflurane, the rats were placed on an isothermal heat pad. Hair above the abdomen and perineum was shaved using hair clippers for better visualization. A 2.5-cm lower abdominal midline incision was used to identify and expose the major pelvic ganglia (MPG) and the CNs. The CNs were isolated bilaterally and crushed 5 mm distal to the MPG of 90s using micro-forceps (Storz, Germany). Then, the CNI group was randomly divided into three groups of 12 rats each, which received cavernous injection of 1) ASCs (1 × 106 cells in PBS 0.1 ml); 2) CBMSCs (1 × 106 cells in PBS 0.1 ml); and 3) PBS (PBS 0.1 ml). The MSCs were injected into the corpus cavernosum immediately after CNI. The injection method was performed as previously described (Fang et al., 2018). The penis was locally exposed, and a rubber tourniquet was applied to the base. After 0.1 ml PBS containing 106 cells was injected into the corpus cavernosum, the tourniquet was released after 1 min, and the penis was restored.

### Erectile function evaluation

The maximal intracavernous pressure (ICP) and real-time arterial pressure were recorded as previously described (Chen et al.). In brief, 4 weeks or 12 weeks after injection, the rats were anesthetized by pentobarbital sodium (40 mg/kg). After anesthesia, the abdomen was incised; the intact penis and prostate were exposed. After locating the MPGs and CNs, a 26-gauge needle connected to a catheter was inserted into the corpus cavernosum to measure ICP, while the other end of the catheter was connected to a data collection device (BL-420s, Chengdu Taimeng Software Co. Ltd., China) using a pressure transducer. After exposing the left carotid artery, a 20-gauge cannula filled with heparin saline was punctured in the artery to measure the mean artery pressure (MAP). The other end of the cannula was also connected to the BL-420s using a pressure transducer. A stimulatory electrode was placed under the cavernous nerve for stimulation. The stimulus parameters were set at 6 V, 25 Hz, and 60 s duration. For each rat, the ratio of maximal ICP to MAP (ICP/MAP) was calculated to compare the erectile function. After acquiring ICP and MAP data, the penis was excised for further testing, and then the rats were euthanized with carbon dioxide.

### Histological and immunoflourescence analysis

For Masson trichrome staining, the tissues harvested from the rats were fixed in 4% paraformaldehyde overnight; then, the tissues were dehydrated and embedded in paraffin. Next, the paraffin-embedded tissues were cut into 4-um sections for staining. After gradient dehydration with xylene alcohol, the tissues were stained with the Masson trichrome stain kit (Masson Trichrome Stain Kit, Solarbio, United States). The stained sections were observed under the microscope and analyzed using Image J.

For immunofluorescence analysis, fresh tissues were harvested and immersed in 4% paraformaldehyde for fixation. Then, the tissues were immersed in 30% sucrose PBS solution for dehydration. After being embedded in OCT, the paraffin-embedded tissues were cut into sections, the sections were blocked with goat serum (Sigma-Aldrich, MO, United States) for 1 h at room temperature, and then incubated with the following primary antibodies: anti-desmin antibody (1:200, ab32575, Abcam), anti-nNOS antibody (1:100, ab177487, Abcam), anti-α-SMA antibody (1:200, #19245, CST), and anti-CD31 antibody (1:100, ab222783, Abcam). Thereafter, the sections were incubated with the secondary antibody (Millipore, MA, United States) for 60 min at room temperature. Six fields were captured randomly in each section. The photographs were analyzed using Image J software. Results were presented as the ratio of immunofluorescence positive area *versus* the total area.

### Intracellular ROS detection

Schwann cells were obtained from the Shanghai Binhui Biotechnology Company (S16 Adherent). Intracellular reactive oxidative species (ROS) were measured by flow cytometry using an oxidation-sensitive fluorescent probe, DCFH-DA (S0033, Beyotime, China), which is oxidized to DCF in the presence of ROS. Schwann cells cultured in six-well plates were incubated with 1 μmol/L DCFH-DA at 37°C for 20 min Then, the cells were treated with ASC-CM or CBMSC-CM for 6 h. Subsequently, 5 μmol/L H_2_O_2_ solution was added to the supernatants to mimic oxidative conditions. DCF fluorescence was detected by CytoFLEX (Beckman Coulter, United States).

### Mitochondrial membrane potential assay

The JC-1 probe (Beyotime, China) was employed to measure mitochondrial membrane potentials in Schwann cells. Briefly, cells were cultured in six-well plates stained with JC-1 staining solution, then incubated with ASC-CM or CBMSC-CM for 6 h, and then 5 μmol/L H_2_O_2_ solution was added to the supernatants. Mitochondrial membrane potentials were detected by the relative amounts of dual emissions from mitochondrial JC-1 monomers or aggregates using the confocal laser scanning microscope (Leica, Germany).

### EdU cell proliferation staining

EdU cell proliferation staining was performed using an EdU kit (BeyoClick™ EdU Cell Proliferation Kit with Alexa Fluor 488, Beyotime, China). Schwann cells were seeded in a six-well plate and treated with ASC-CM or CBMSC-CM for 6 h, followed by H_2_O_2_ stimulation for 2 h. Then, cells were incubated with EdU and Hoechst 33,342 according to the manufacturer's instructions. The fluorescence was detected using the confocal laser scanning microscope (Leica, Germany).

### Cell apoptosis assay

Annexin V-FITC/PI cell apoptosis detection kit (Yeasen Biology, China) was used to detect the level of apoptosis under different conditions. Schwann cells seeded in a six-well plate (costar, United States) were pre-treated with ASC-CM or CBMSC-CM for 6 h and then stimulated with 5 μmol/L H_2_O_2_ for 2 h. Subsequently, the cells were collected and stained with Annexin V-FITC and PI probe solution at room temperature for 15 min. The apoptosis was detected by Cytoflex (Beckman Coulter, United States).

### Analysis of soluble factors in the CBMSC-CM and ASC-CM

To determine the soluble factors in CBMSCs-CM and ASCs-CM, a protein antibody array was performed with a RayBiotech L-series human Antibody Array 507. The CM of hEF was used as a control. The experiment procedure was conducted according to the manufacturer’s instructions. Finally, the fluorescent signals on the glass slide were scanned using GenePix 4000B (Axon Instruments).

### ELISA analysis

The NT4, VEGF, MMP1, and MMP3 levels in the CBMSC-CM and ASC-CM were measured using an ELISA kit of NT4 (Invitrogen, United States), VEGF (MULTI SCIENCES, China), MMP1 (MULTI SCIENCES, China), and MMP3 (MULTI SCIENCES, China) according to the manufacturer's instruction. The absorbance was detected at 450 nm using a BioTek Gene 5 automated Microplate Reader (Bio-Tek Instruments).

### Statistical analysis

All quantitative data were expressed as the mean ± standard deviation. Differences between the groups were assessed by one-way analysis of variance (ANOVA), followed by a Student’s t-test using GraphPad Prism v7.0 software. *p* < 0.05 was considered statistically significant.

## Results

### Characterization of CBMSCs and ASCs

**FIGURE 1:**
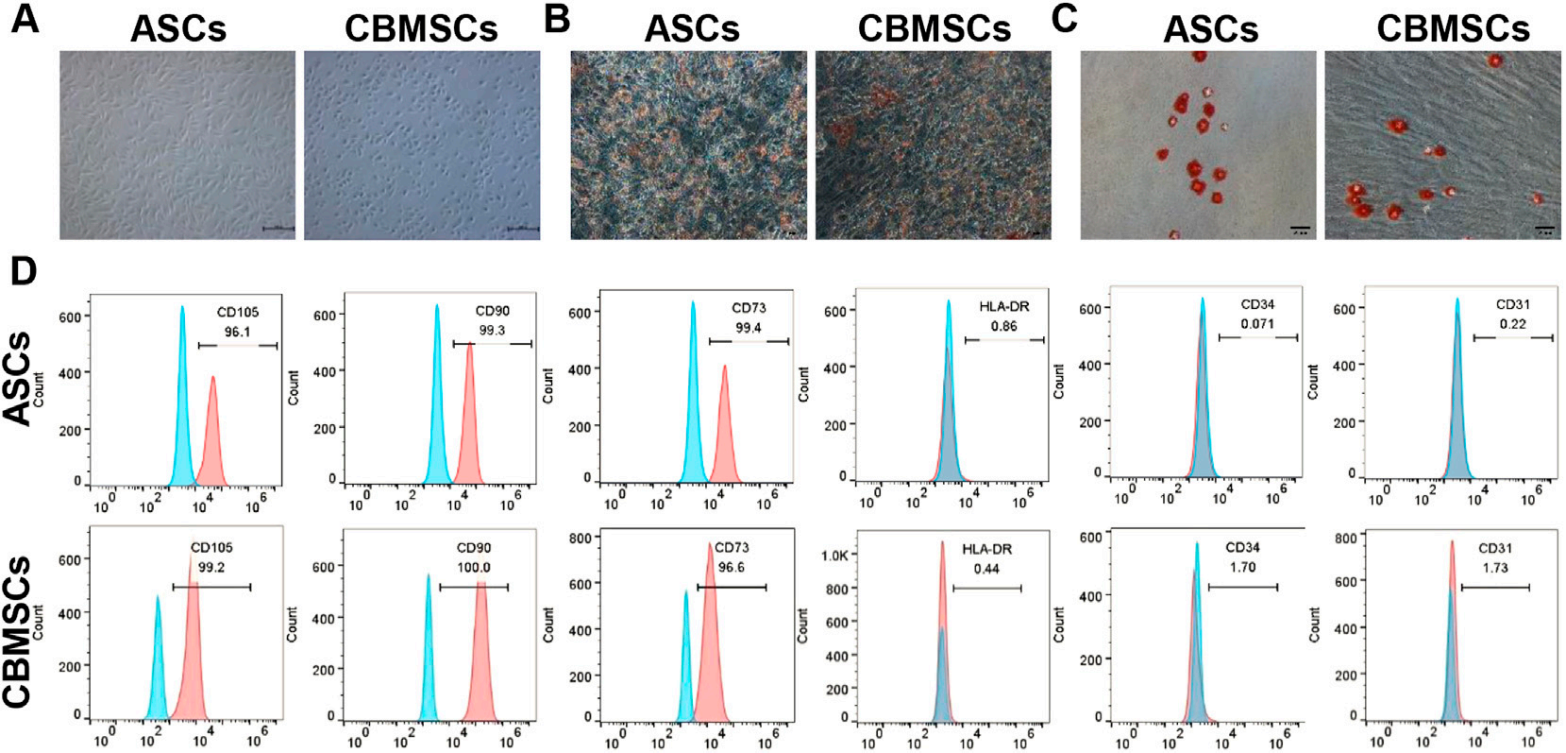
Identification of CBMSCs and ASCs. **(A)** Morphology of CBMSCs and ASCs. **(B)** Red-O staining of CBMSCs and ASCs after adipogenic induction for 21 days. **(C)** Alizarin Red staining of CBMSCs and ASCs after osteogenic induction for 14 days. **(D)** Surface markers (CD105, CD90, CD73, CD45, and CD31) of CBMSCs and ASCs were identified by flow cytometry.

Primary CBMSCs and ASCs were isolated from umbilical cord blood and adipose tissues according to the protocol in the Methods and materials section. Both CBMSCs and ASCs exhibited spindle-shaped morphology (Figure 1A). To determine the differentiation potential of CBMSCs and ASCs, *in vitro* adipocytic and osteoblastic differentiation assays were performed. The results showed that CBMSCs and ASCs were positive with Oil Red O staining after adipogenic induction (Figure 1B) and positive with Alizarin staining after osteogenic induction (Figure 1C). In addition, the surface markers of CBMSCs and ASCs detected by flow cytometry indicated that both CBMSCs and ASCs expressed classical MSC markers, including the positive expression of CD73, CD90, and CD105 and negative expression of CD45, CD31, and HLA-DR (Figure 1D). Collectively, these findings confirmed the characteristics of *in vitro* expanded cells as CBMSCs and ASCs.

### CBMSC treatment restored erectile function in the CNI rat model

**FIGURE 2:**
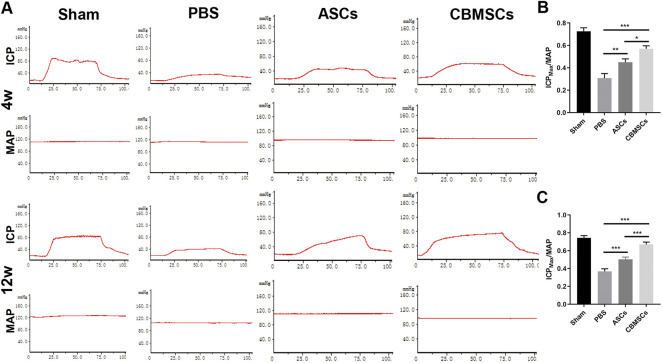
ICP/MAP of rats in different groups. **(A)** Injection of MSCs significantly restored the erectile function of CNI-ED rats at 4 weeks and 12 weeks after IC injection; the representative images of ICP and MAP after CN electrical stimulation are shown. The statistical analysis of maximal ICP to MAP (ICPmax/MAP) in 4 weeks **(B)** and 12 weeks **(C)** are recorded.

To evaluate the effect of CBMSC transplantation on the recovery of erectile function, the ratio of the maximum ICP to MAP (ICPmax/MAP) was measured as shown in Figure 2A. Compared with the sham group, ICPmax/MAP of the PBS group was remarkably decreased, indicating that erectile function was impaired after bilateral cavernous nerve injury. However, ICPmax/MAP of CBMSC and ASC groups was significantly improved when compared with the PBS group, which improved to 86% and 66% of that in the sham group, respectively (Figures 2B,C). Moreover, ICPmax/MAP of the CBMSC group was apparently higher than that of the ASC group (*p* < 0.05), demonstrating that CBMSCs were superior to ASCs in the preservation of erectile function.

To investigate the long-term effect of CBMSC treatment on erectile function, we assessed the functional recovery in 12 weeks after injection. Similar to 4 weeks, ICPmax/MAP of the PBS group, ASC group, and CBMSC group was still lower than that of the sham group. ASC and CBMSC groups were better than the PBS group. However, compared with 4 weeks, the ASC group and CBMSC group all increased slightly, but the recovery of the CBMSC group was still better than that of the ASC group (*p* < 0.001).

### CBMSC treatment restored smooth muscle ingredient and increases nNOS expression

**FIGURE 3:**
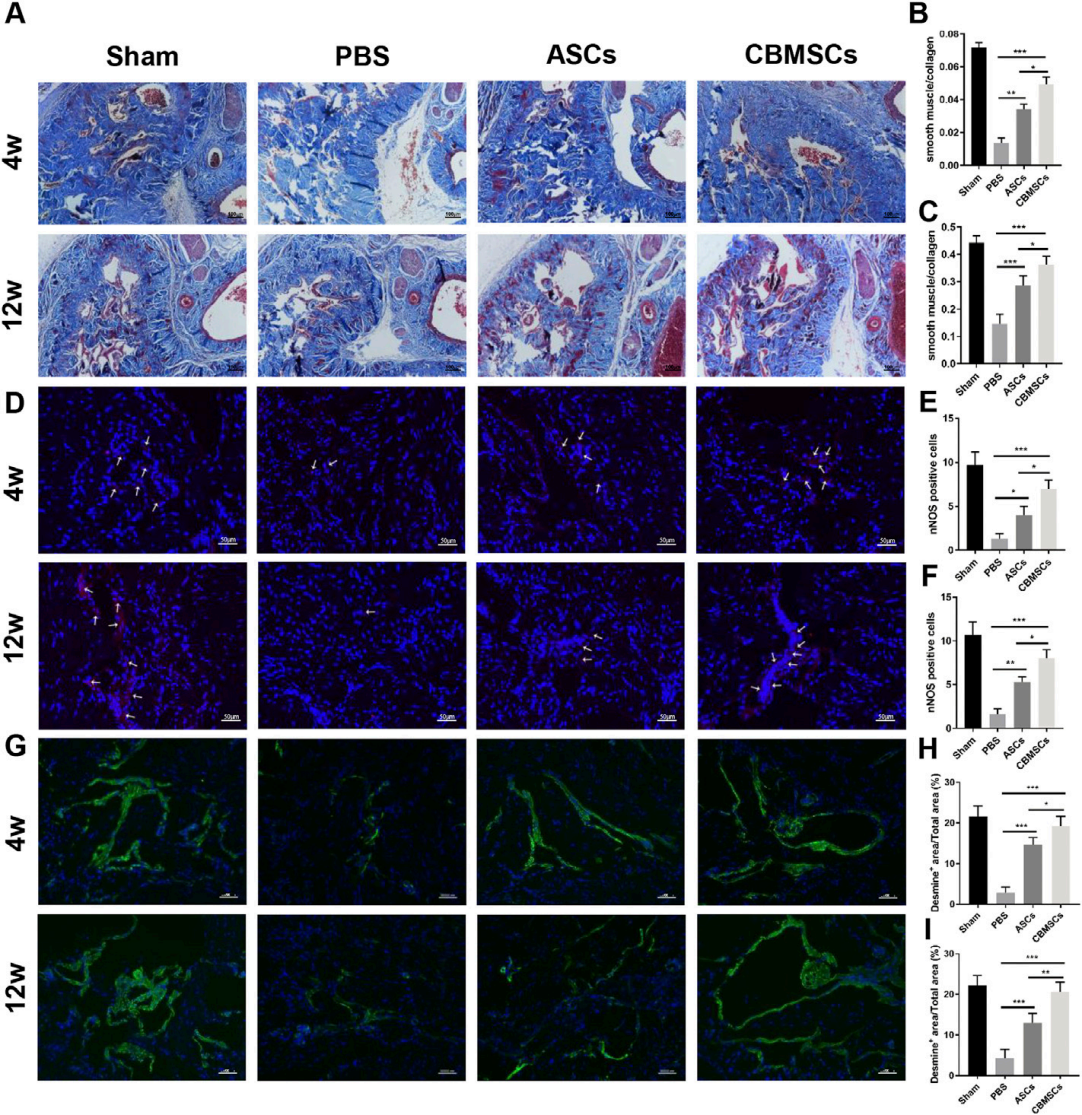
Masson trichrome staining and immunofluorescence staining of rat corpus cavernosum. **(A)** Representative images of Masson trichrome staining of the corpus cavernosum in a penile midshaft specimen. Magnification: ×100. Statistical analysis of Masson trichrome staining of rat corpus cavernosum at 4 weeks **(B)** and 12 weeks **(C)**. **(D)** Representative images of immunofluorescence staining of nNOS of rat corpus cavernosum. Red arrowheads: nNOS-positive area. Statistical analysis of immunofluorescence staining of nNOS at 4 weeks **(E)** and 12 weeks **(F)** after treatment. **(G)** Representative images of immunofluorescence staining of desmin of rat corpus cavernosum. Statistical analysis of immunofluorescence staining of desmin at 4 weeks **(H)** and 12 weeks **(I)**. *p < 0.05, **p < 0.01, and ***p < 0.001.

Masson trichrome staining was used to assess the smooth muscle and collagen content as shown in Figure 3A. Four weeks after injection, the ratio of smooth muscle to collagen was significantly decreased in the PBS group when compared with the sham group, whereas CBMSC and ASC administration rescued the loss of cavernous corporal smooth muscles (Figures 3B,C). Similarly, 12 weeks after injection, the ratio of smooth muscle to collagen in the PBS group was remarkably decreased when compared to the sham group, and CBMSC or ASC administration attenuated the decrease in the ratio. Of the two cell groups, the improvement in the CBMSC group was significantly better than that of the ASC group. Subsequently, we used immunofluorescent staining of desmin, a specific marker of cavernous smooth muscle, to further evaluate the restoration of the smooth muscle content. As shown in Figures 3G–I, after 4 weeks, the positive area of desmin in the PBS group, CBMSC group, and ASC group was lower than that in the sham group. The ASC group and CBMSC group showed certain recovery compared with the PBS group, and the CBMSC group showed better recovery than the ASC group (*p* < 0.05). Similar trends were seen 12 weeks after injection, and the CBMSC group still showed better recovery than the ASC group (*p* < 0.01). These data revealed the efficiency of CBMSCs in protecting CCSMC content and reducing collagen deposition.

To explore the effect of CBMSC application on the repair of cavernous nerve, we detected nNOS expression, a specific marker of cavernous nerve, by immunofluorescence staining. As shown in Figures 3D–F, after 4 weeks, the intensity of nNOS fluorescence in the corporal cavernous of the PBS group, ASC group, and CBMSC group was lower than that in the sham group; however, compared with the PBS group, ASC and CBMSCs groups showed certain recovery. Moreover, the CBMSC group exhibited better recovery than the ASC group (*p* < 0.05). After 12 weeks, therapeutic application of CBMSCs and ASCs profoundly mitigated the decrease of nNOS expression, and this effect mediated by CBMSC injection was much better than that by ASC injection (*p* < 0.05).

### CBMSCs showed limited differentiation potential in the CNI-ED rat model

**FIGURE 4:**
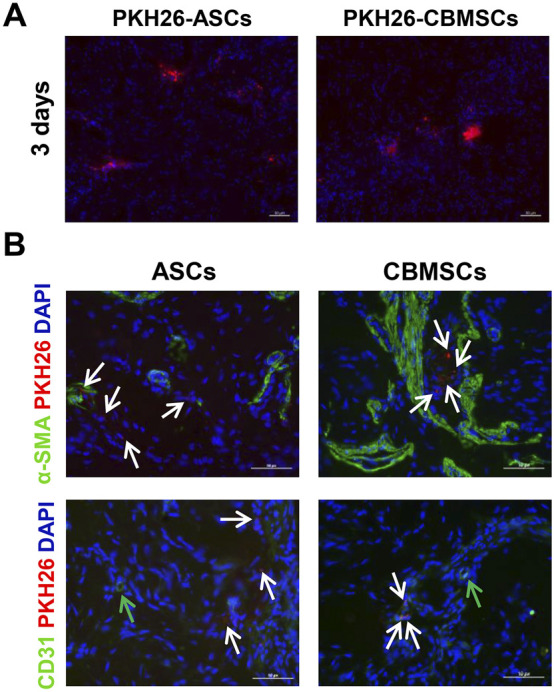
CBMSCs showed poor differentiation potential in the penis. **(A)** PKH-26-labeled CBMSCs were detected at 3 days after intracavernosal injection in CNI-ED rats. **(B)** Differentiation potential of CBMSCs was assessed by colocalization with the CCSMC marker and CD31 marker. White arrows: PKH-26-labeled CBMSCs or ASCs. Green arrows: CD31-positive endothelial cells.

To determine the effect of CBMSC differentiation on the restoration of erectile function, CBMSCs and ASCs were labeled with PKH-26 and then injected into the corpus cavernosum of CNI-ED rats. The presence and differentiation potential of CBMSCs was detected by immunofluorescence staining. Three days after injection, a number of CBMSCs and ASCs were observed in the corpus cavernosum, as indicated by the red fluorescent signal (Figure 4A). Considering that CCSMCs and endothelial cells (ECs) were the two main cell types in the corpus cavernosum and played a critical role in normal erectile function of the penis (Shamloul and Ghanem, 2013), the differentiation potential of CBMSCs into CCSMCs and ECs was accessed. The immunofluorescence staining data indicated that PKH-26-labeled CBMSCs and ASCs were not colocalized with CCSMC marker α-SMA and EC marker CD31 (Figure 4B), suggesting the poor differentiation potential of CBMSCs and ASCs in the penis and other mechanisms mediating the recovery of erectile function.

### CBMSC secretomes mitigated oxidative stress and apoptosis and promoted cell proliferation

**FIGURE 5:**
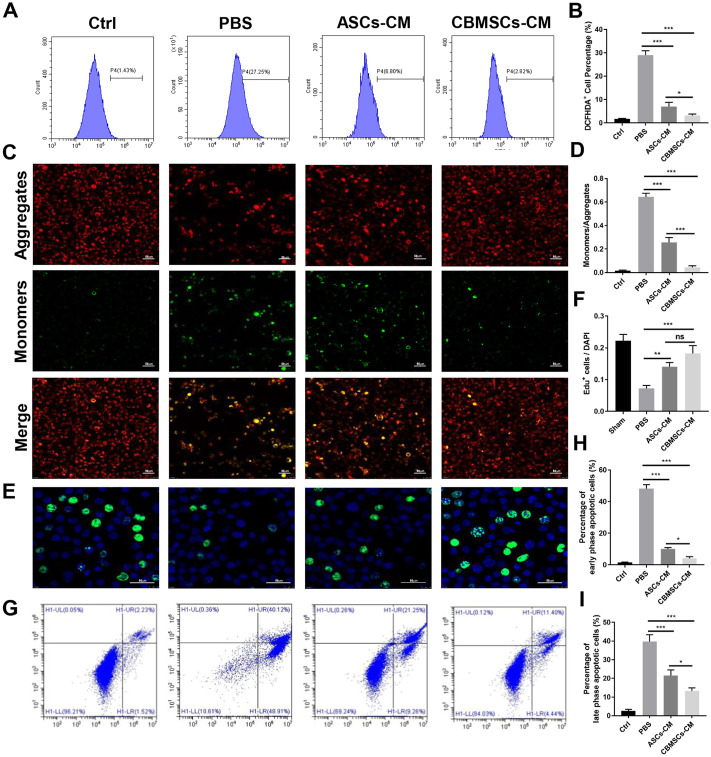
Effects of CBMSC-CM and ASC-CM on Schwann cells and CCSMCs *in vitro*. **(A)** Intracellular ROS in Schwann cells were detected by flow cytometry after treatment with CBMSC-CM or ASC-CM and stimulation with H_2_O_2_. **(B)** Statistical analysis of ROS in Schwann cells. **(C)** Schwann cells in an early apoptotic state were assessed by mitochondrial membrane potential staining. Representative images are shown. **(D)** Statistical analysis of Schwann cells in the early apoptotic state. **(E)** Proliferating Schwann cells were measured by EdU staining after CBMSC-CM or ASC-CM incubation and H_2_O_2_ stimulation. **(F)** Statistical analysis of EdU-positive Schwann cells. **(G)** Apoptosis of CCSMCs was examined by flow cytometry after CBMSC-CM or ASC-CM treatment and H_2_O_2_ exposure. **(H)** Statistical analysis of early-stage apoptotic CCSMCs. **(I)** Statistical analysis of early-stage apoptotic CCSMCs. *p < 0.05, **p < 0.01, and ***p < 0.001.

To further investigate the protective role of CBMSCs in cavernous nerve repair, we collected the conditioned medium (CM) of CBMSCs and ASCs and pre-treated the rat primary Schwann cells with CM for 6 h followed by H_2_O_2_ exposure for 2 h. After H_2_O_2_ treatment, the ROS level of the Schwann cells obviously increased compared with that of the control group as shown in Figures 5A,B. Nevertheless, the ROS level in both CM groups decreased remarkably, and it was lower in the CBMSC-CM group than ASC-CM group, indicating that the active components existed in CM of CBMSCs possessed a better anti-oxidative stress effect compared with those in ASC-CM (*p* < 0.05).

Exposure of Schwann cells to H_2_O_2_ for 2 h resulted in mitochondrial depolarization, which is shown as increased green fluorescence and decreased red fluorescence (Figures 5C,D). The mitochondrial depolarization was both moderated in Schwann cells treated with ASC-CM or CBMSC-CM, as shown by decreased green fluorescence and increased red fluorescence, suggesting that CBMSC-CM had a better anti-apoptotic effect than ASC-CM (*p* < 0.01).

Next, we performed EdU staining assay to determine the effect of CBMSCs secretomes on Schwann cell proliferation. EdU-positive cells were stained green, and the nuclei were stained blue by DAPI. Exposure of Schwann cells to H_2_O_2_ for 2 h resulted in inhibition of proliferation of Schwann cells compared with the sham group (Figures 5E,F). The proliferation rate of Schwann cells increased after being cultured with ASC-CM or CBMSC-CM, and CBMSC-CM treatment showed a higher improvement than ASC-CM treatment (*p* < 0.05).

In addition, we assessed the protective role of CBMSC secretions on rat cavernous smooth muscle cell (CCSMCs) apoptosis. After exposed to H_2_O_2_ for 2 h, the percentage of early-phase and late-phase apoptotic CCSMCs increased significantly than that of the control group (Figures 5G–I). Conversely, the percentage of early-phase and late-phase apoptotic cells in the ASC-CM group or CBMSC-CM group decreased substantially, and both early-phase and late-phase apoptotic cells in the CBMSC-CM group were significantly less than those in the ASC-CM group (*p* < 0.05, *p* < 0.05). These findings indicated that CBMSC secretomes are superior to ASC-CM in alleviating apoptosis.

### CBMSC secretomes are rich in neurotrophic factors and matrix metalloproteinases

**FIGURE 6:**
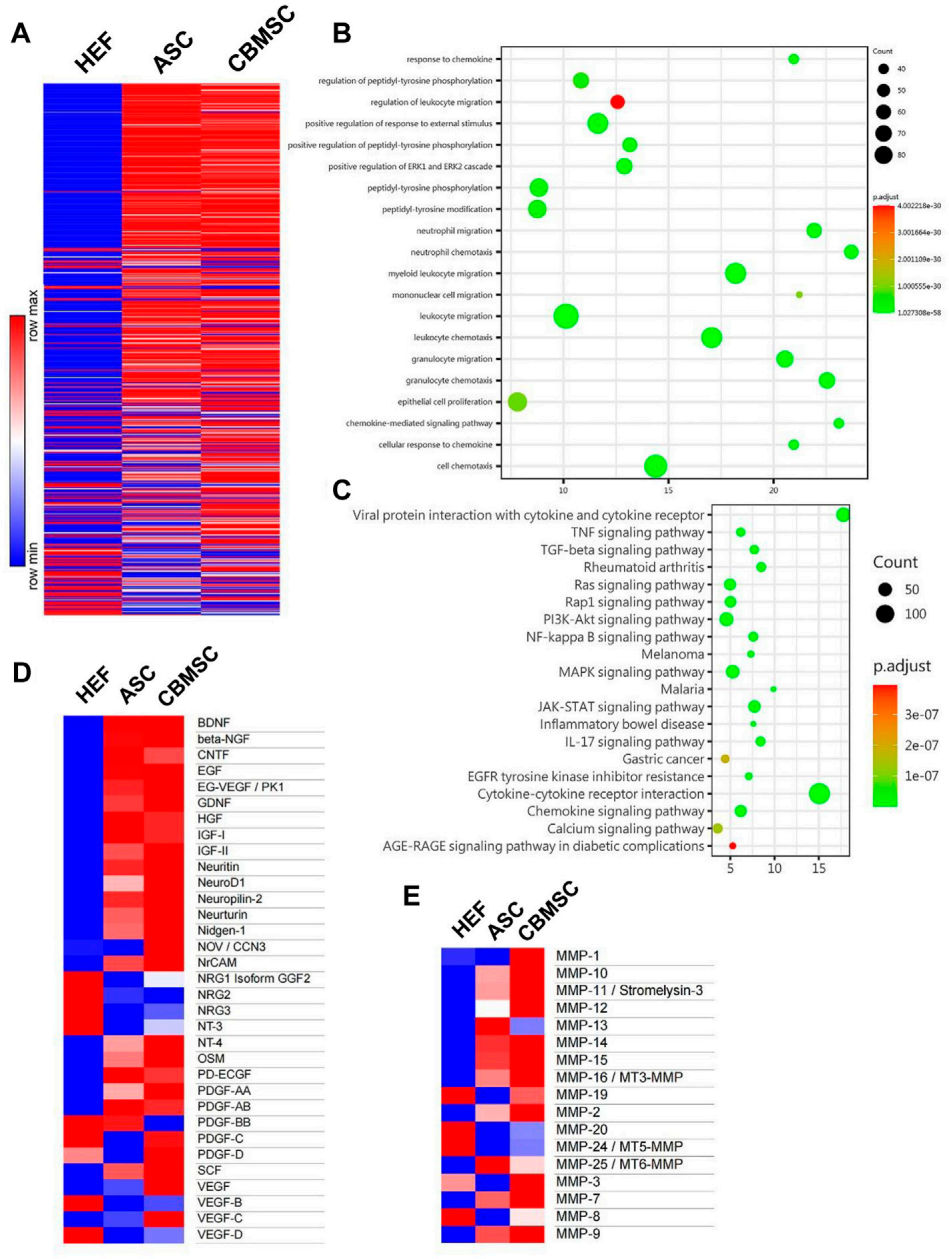
Soluble factors secreted by CBMSCs and ASCs in the conditioned medium were analyzed using protein chips. **(A)** Relative concentration of 507 proteins in HEF-CM, ASC-CM, and CBMSC-CM is shown in a heat map. **(B)** Gene Ontology (GO) function enrichment analysis of biological processes of CBMSC-CM vs. ASC-CM. **(C)** KEGG signaling pathway enrichment analysis of CBMSC-CM vs. ASC-CM. Heat map of differentially expressed neurotrophic factors **(D)** and matrix metalloproteinases **(E)** in CBMSC-CM vs. ASC-CM. See also Supplementary Tables S1–S3. hEF, CBMSC, and ASC represent the CMs of these cells.

Substantial compelling evidence has substantiated that MSCs exerted their function mainly through a paracrine mechanism (Gnecchi et al., 2008; Gnecchi et al., 2016). Therefore, to further investigate the underlying mechanism of CBMSC treatment on cavernous nerve repair and cavernous smooth muscle restoration, we analyzed CBMSC secretory factors by a human cytokine antibody array 507. The CM of hEF was used as a control. As shown in Figure 6A and Supplementary Table S1, the relative expression levels of the majority of soluble factors in the CM of CBMSCs were much higher than those of ASCs or hEF. Subsequently, we performed Gene Ontology (GO) function enrichment analysis of biological processes of CBMSCs vs. ASCs. We found that secretion factors related to response to chemokine, regulation of peptidyl-tyrosine phosphorylation, and regulation of leukocyte migration in the CBMSCs were significantly upregulated compared to ASCs (Figure 6B). KEGG enrichment analysis demonstrated that several pathways related to the Ras signaling pathway, PI3K-Akt signaling pathway, and JAK-STAT signaling pathway were activated in CBMSCs (Figure 6C). Moreover, further analysis of neurotrophic factors revealed that CBMSCs secreted much higher growth factors related to nerve repair and growth, including neuritin, NeuroD1, neuropilin-2, neurturin, nidogen-1, NrCAM, NT4, and NT3 (Figure 6D; Supplementary Table S2). Furthermore, we also found that most matrix metalloproteinase (MMP) activities involved in extracellular matrix degradation in the CM of CBMSCs were much higher than those of ASCs (Figure 6E; Supplementary Table S3), which was consistent with the lower fibrosis degree observed in the CBMSC group (Figure 3A). Taken together, this evidence indicated that CBMSC secretomes are the basis for exerting a better therapeutic effect on CNI-ED.

### Quantification of secretory factors from CBMSC secretomes

**FIGURE 7:**
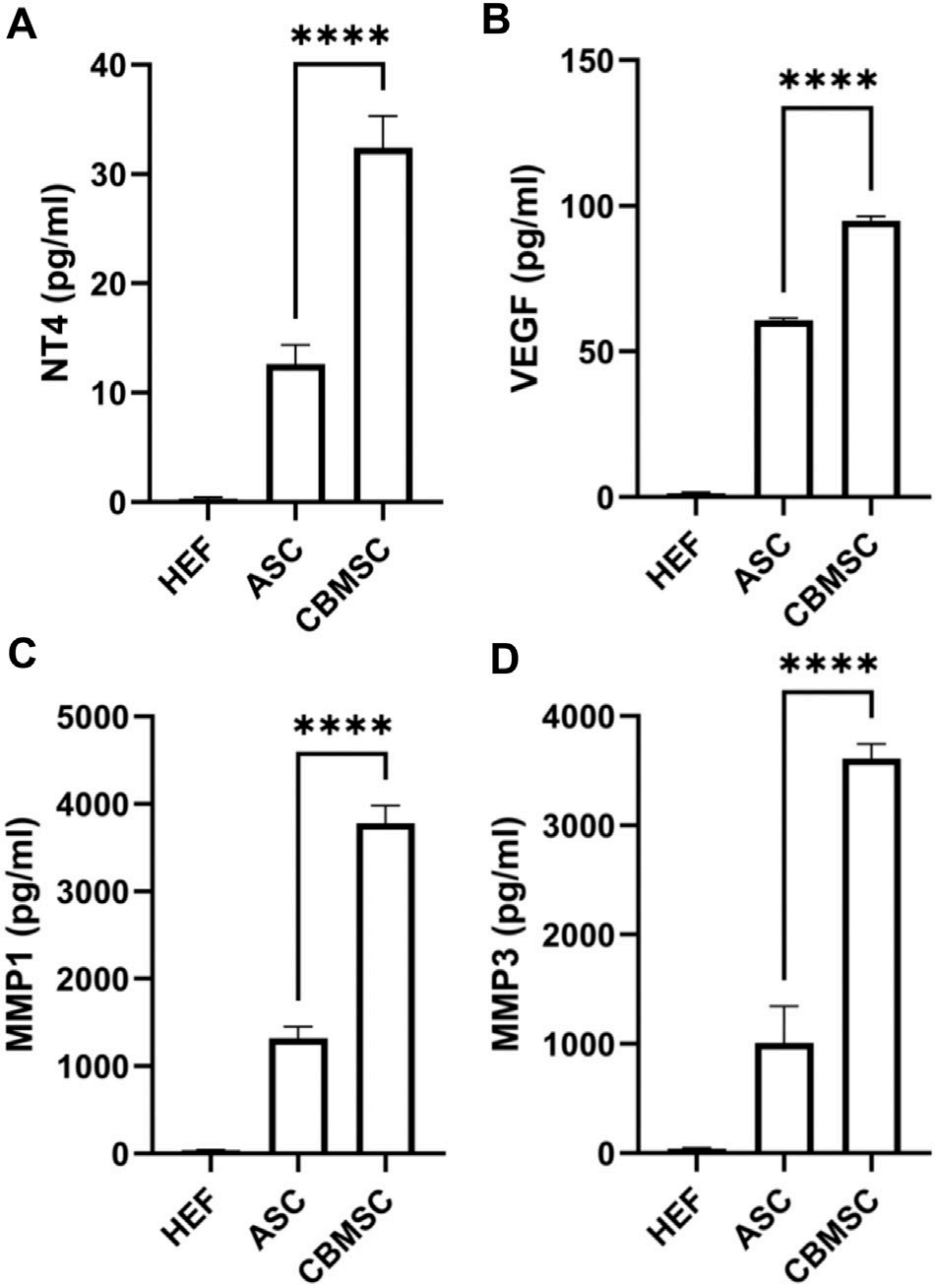
Quantitative analysis of NT4, VEGF, MMP1, and MMP3 levels of CBMSC-CM. The levels of NT4 **(A)**, VEGF **(B)**, MMP1**(C)**, and MMP3 **(D)** from HEF-CM, ASC-CM, and CBMSC-CM were detected by ELISA assay.

Previous studies have reported that growth factors including BDNF, β-NGF, GDNF, neurturin, IGF, NT3, NT4, and VEGF (Hannan et al., 2015; Takayanagi et al., 2015; Yang W. et al., 2020) and matrix metalloproteinases including MMP1, MMP3, MMP8, MMP9 (Yang Q. et al., 2020; Sturny et al., 2021) contributed to the erectile function recovery, and thus we quantified the NT4, VEGF, MMP1 and MMP3 levels (some differentially expressed factors) in the CM of CBMSCs and ASCs using the ELISA kit. According to the quantification data, the level of NT4 and VEGF in CBMSC-CM was significantly higher than that in the ASC-CM group (*p* < 0.001) (Figures 7A,B). Moreover, the level of MMP1 and MMP3 was also significantly higher in the CBMSC-CM group than the HEF and ASC-CM group (*p* < 0.001) (Figures 7C,D).

## Discussion

ED is defined as the failure to achieve an erection sufficient for penetration (Shamloul and Ghanem, 2013). It is estimated that 5%–10% of 40-year-old men are affected (Inman et al., 2009). Among them, CNI-ED patients are extremely hard to treat in clinics. Owing to cavernosum fibrosis after nerve injury, CNI-ED patients hardly show a response to conventional therapies such as PDE-5 inhibitors (Hatzimouratidis et al., 2009). Stem cell therapy offers promising future for CNI-ED in addition to conventional therapies. Several types of stem cells have been applied to restore erectile function of the CNI-ED rats (Chen et al., 2017; Sun et al., 2017; Fang et al., 2018; Chen et al., 2019), and the therapeutic effects vary with cell origins (Gu et al., 2019). ASCs are widely used in the field of tissue engineering and have been applied to restore the erectile function of diabetic ED rats in our previous study (Yan et al., 2020). CBMSCs are isolated from cord blood of the newborn which has lower immunogenicity. CBMSCs are more primitive than other adult MSCs and are readily available due to the extensive establishment of cord blood banks.

In our research, the efficacy of ASCs and CBMSCs was studied at 4 weeks and 12 weeks after intra-cavernosum injection. Both ASC and CBMSC groups showed significant recovery of erection function and morphology restoration, and the CBMSC group exhibited better functional and histological results than the ASC group. In addition, previous studies have demonstrated that Schwann cells (SCs) enhance the regeneration of erectile nerves and facilitated the recovery of erectile function in BCNI rats (May et al., 2008). Oxidative stress occurs immediately in the corpus cavernosum after CN injury (Wu et al., 2018), and H_2_O_2_ was applied to induce CCSMC apoptosis in the previous study (Ouyang et al., 2018). Thus, SCs exposed to H_2_O_2_ were used to simulate cavernous nerve damage, and the protective effect of CBMSC-CM on SCs was determined by *in vitro* experiments. These *in vitro* experiments combined with CCSMC apoptosis detection demonstrated the efficiency of CBMSC transplantation on nerve recovery and its superiority to ASCs.

Mechanically, the differentiation capacity of CBMSCs was assessed in CNI-ED rats through immunofluorescence colocalization staining, which indicated that no differentiation occurred within 3 days after the cells were injected into the corpus cavernosum. Numerous studies have illustrated that MSCs exerted their function mainly in a paracrine pattern (Gnecchi et al., 2016). Therefore, we focus on soluble factors secreted by CBMSCs and ASCs. We analyzed the content of secretory factors in the CM of CBMSCs and ASCs by protein chips, suggesting that the majority of neurotrophic factors secreted by CBMSCs were much higher than that of ASCs, especially VEGF and NT4. VEGF is recognized as important growth factors for vascularization and cavernous nerve regeneration. Studies have investigated the delivery of VEGF and BDNF, which exerted synergistic effect to promote cavernous nerve regeneration (Lin et al., 2010; Zhang H. Y. et al., 2011; Zhang et al., 2016), and the activation of the JAK/STAT signaling pathway played a major role in VEGF- and BDNF-mediated MPG growth (Zhang H. Y. et al., 2011). Meng et al. (2019) reported that NT4 signaling regulates cell proliferation, survival, axon and dendrite growth, the fate of neural precursors through the Trk receptor-activating Ras signaling pathway as well as the MAP kinase pathway and PI3-kinase pathway, which were also activated in CBMSCs (Figure 5C). The Ras/Raf/MAPK signaling pathway is a classic signaling pathway, which plays an important role in the transmission of proliferative signals from membrane-bound receptors and in regulating cellular proliferation (Liu et al., 2017). Therefore, in our study, there is a rationale for speculating that VEGF and NT4 promoted the recovery of cavernous nerves by activating JAK/STAT and Ras/Raf/MAPK signaling pathways, thus improving erectile function.

In addition, matrix metalloproteinases (MMPs) in the CM of CBMSCs were much higher than those of ASCs, suggesting stronger anti-fibrotic effects of CBMSCs than those of ASCs. The production of extracellular matrix is most commonly recognized as an important pathological change in the corpus cavernosum of CNI-ED. Moreover, decreased net MMP activity was observed in patients with erectile dysfunction (Muniz et al., 2012), while intratunical injection of MSCs relieved penile fibrosis by stimulating the expression and activity of MMPs (Gokce et al., 2014). MMPs are capable of degrading all kinds of extracellular matrix proteins. In our study, many types of MMPs are upregulated in the CM of CBMSCs compared with ASCs, including MMP-1, MMP-2, MMP-3, MMP-7, MMP-8, MMP-9, MMP-10, MMP-11, MMP-12, and MMP-19, among which MMP1, MMP2, MMP3, MMP8, and MMP9 have been reported to contribute to erectile function recovery (Yang Q. et al., 2020; Sturny et al., 2021; Cohen et al., 2022). Further quantification of MMP1 and MMP3 by ELISA suggested that CBMSC-CM was nearly three times higher than ASC-CM. These MMPs synergistically boost the degradation of extracellular matrix and alleviate corpus cavernosum fibrosis.

It is reported that the therapeutic potential of MSCs depends not only on the paracrine effect on adjacent cells but also on the stem cell niche and autocrine mechanism to enhance their own proliferation and survival after injection (Sid-Otmane et al., 2020). For example, a previous study demonstrated that autocrine FGF-2 and HGF were important to the stemness of MSCs (Eom et al., 2014). Autocrine production of PDGF promotes the proliferation of neural progenitor cells (Erlandsson et al., 2006). In addition, VEGF signaling has been shown to modulate hematopoietic stem cell survival *via* an internal autocrine loop (Gerber et al., 2002). Furthermore, autocrine IGF improves stem cell-mediated neuroprotection through increasing GDNF production and accelerating neurite outgrowth (Lunn et al., 2015). Among these autocrine factors, VEGF, PDGF, and IGF contents in CBMSC-CM was significantly higher than those in ASC-CM (Figure 6C). Taken together, these autocrine factors facilitated maintenance of the viability and therapeutic potential of CBMSCs and ASCs.

There are some limitations to our study. First, there is a lack of experiments on the effectiveness of injecting CBMSC-CM and ASC-CM into the corpus cavernosum. Second, although we have identified and quantified some differentially expressed soluble factors of CBMSCs, a further study aimed at elucidating the downstream signaling pathways will be performed. Third, exosomes secreted by MSCs have been demonstrated to be beneficial to CNI-ED (Ouyang et al., 2018); hence, further research needs to be performed to elucidate the role of exosomes in the paracrine effect of CBMSCs and ASCs.

## Conclusion

Intracavernous injection of CBMSCs exhibited a better erectile function restoration than that of ASCs in CNI-ED rats, which might be due to richer neurotrophic factors and MMPs secreted by CBMSCs. CBMSC transplantation may be a promising therapeutic strategy for treating CNI-ED in the future.

## Data Availability

The original contributions presented in the study are included in the article/Supplementary Material; further inquiries can be directed to the corresponding authors.
