# Supplementary material for: Comparison of the therapeutic effects of human umbilical cord blood-derived mesenchymal stem cells and adipose-derived stem cells on erectile dysfunction in a rat model of bilateral cavernous nerve injury
Source: Front Bioeng Biotechnol. 2022 Oct 7;10:1019063. doi: 10.3389/fbioe.2022.1019063 (PMC9585154; doi:10.3389/fbioe.2022.1019063)
Supplement: Supplementary file 1 [file Table1.DOCX]

Table S1: A table of normalized fluorescence signal intensity of 507 soluble factors in the CMs of hEF, ASCs and CBMSCs. hEF represents the CM of hEF, ASC represent the CM of ASCs, CBMSC represent the CM of CBMSCs.

| proteinID | HEF | ASC | CBMSC |
| --- | --- | --- | --- |
| 6Ckine | 6.826314025 | 12.35979379 | 12.73147814 |
| Activin A | 10.50779424 | 13.18465877 | 12.26224169 |
| Activin B | 0.0581297 | 12.71361948 | 12.7995346 |
| Activin C | 7.306057213 | 10.82526148 | 10.91550596 |
| Activin RIA / ALK-2 | 6.852545566 | 11.9051805 | 11.91578603 |
| Activin RIB / ALK-4 | 6.074937988 | 12.03307307 | 11.75154406 |
| Activin RII A/B | 1.865484622 | 10.02945981 | 6.199672345 |
| Activin RIIA | 0.0581297 | 12.17562207 | 11.97575842 |
| Adiponectin / Acrp30 | 3.145592541 | 11.70097594 | 11.44643718 |
| AgRP | 9.000644205 | 11.02287703 | 10.75279876 |
| ALCAM | 2.306057213 | 12.47756547 | 11.61079417 |
| Angiogenin | 5.627985308 | 10.89215142 | 11.83289001 |
| Angiopoietin-1 | 6.01232601 | 12.04442831 | 11.98886217 |
| Angiopoietin-2 | 10.50650416 | 12.91585255 | 12.76668418 |
| Angiopoietin-4 | 0.0581297 | 11.10496348 | 11.4677329 |
| Angiopoietin-like 1 | 9.761168089 | 12.10649446 | 11.87862573 |
| Angiopoietin-like 2 | 6.775806122 | 11.04920541 | 11.83506349 |
| Angiopoietin-like Factor | 0.005919507 | 9.215533365 | 8.442943496 |
| Angiostatin? | 9.21737035 | 12.46554806 | 12.54133866 |
| APJ | 8.018131632 | 9.870846635 | 9.252665432 |
| APRIL | 6.074937988 | 11.94103393 | 12.15703107 |
| AR (Amphiregulin) | 2.450447123 | 9.922399604 | 10.42626475 |
| Artemin | 8.038269278 | 11.03431249 | 10.47699929 |
| Axl | 0.005919507 | 10.41636941 | 10.37123213 |
| B7-1 /CD80 | 5.52573525 | 12.0050351 | 11.96072599 |
| BACE-1 | 8.092928662 | 9.746052062 | 8.779719355 |
| BAFF R / TNFRSF13C | 3.517561319 | 9.287746785 | 8.667111542 |
| Bax | 8.583650509 | 9.275133468 | 8.422064766 |
| BCMA / TNFRSF17 | 0.005919507 | 10.21747591 | 11.1283166 |
| BD-1 | 10.91338753 | 12.4287595 | 12.34137983 |
| BDNF? | 2.643092201 | 13.45533821 | 13.52930853 |
| beta-Catenin | 1.643092201 | 9.070099379 | 9.614709844 |
| beta-NGF | 2.380057795 | 12.892435 | 13.21219237 |
| BIK | 8.029673254 | 11.78625298 | 11.54061241 |
| BLC / BCA-1 / CXCL13 | 6.509340812 | 10.98009767 | 10.29892174 |
| BMP-15 | 9.761168089 | 11.30825573 | 11.08248173 |
| BMP-2 | 6.846032259 | 12.06472488 | 12.33168674 |
| BMP-3 | 10.25227094 | 12.53757356 | 12.06229916 |
| BMP-3b / GDF-10 | 1.758569418 | 8.927394544 | 8.425215903 |
| BMP-4? | 9.291749377 | 9.765394986 | 10.2485206 |
| BMP-5 | 8.887852435 | 10.86770368 | 10.5028318 |
| BMP-6 | 7.648716749 | 11.44085443 | 11.99223026 |
| BMP-7 | 9.033977668 | 11.78247977 | 11.3986101 |
| BMP-8 | 8.364191389 | 12.5766454 | 12.34033694 |
| BMPR-IA / ALK-3 | 3.730555041 | 9.149163247 | 8.946906274 |
| BMPR-IB / ALK-6 | 0.0581297 | 11.88140675 | 11.91615938 |
| BMPR-II | 5.751616657 | 12.27219796 | 12.16364968 |
| BTC | 8.320224545 | 11.49246128 | 11.50158842 |
| Cardiotrophin-1 / CT-1 | 0.005919507 | 9.975228367 | 9.647908074 |
| CCL14 / HCC-1 / HCC-3 | 8.145592541 | 10.98717614 | 10.95927751 |
| CCL28?/ VIC | 8.938478508 | 12.23161753 | 12.66877421 |
| CCR1 | 5.627985308 | 11.85225898 | 12.07246761 |
| CCR2 | 6.371012655 | 12.21168271 | 12.21082352 |
| CCR3 | 8.1964015 | 11.39533674 | 10.94964436 |
| CCR4 | 8.303682406 | 11.37286445 | 11.14863546 |
| CCR5 | 5.389046578 | 11.00472185 | 11.15860969 |
| CCR6 | 0.0581297 | 11.7600245 | 11.52343944 |
| CCR7 | 7.301303683 | 13.32605489 | 13.034713 |
| CCR8 | 3.758569418 | 13.76570345 | 13.38417665 |
| CCR9 | 0.0581297 | 12.70480319 | 12.59589849 |
| CD 163 | 7.43099476 | 12.19707575 | 12.05333199 |
| CD14 | 3.01232601 | 12.02801562 | 12.29383084 |
| CD27 / TNFRSF7 | 1.758569418 | 12.67118835 | 12.54188311 |
| CD30 / TNFRSF8 | 9.663609218 | 10.78945994 | 10.23062093 |
| CD30 Ligand / TNFSF8 | 6.035409623 | 10.89520086 | 10.98868469 |
| CD40 / TNFRSF5 | 10.11882563 | 12.4525776 | 11.91578603 |
| CD40 Ligand / TNFSF5 /CD154 | 7.561955438 | 12.92356004 | 13.15268096 |
| Cerberus 1 | 6.357337718 | 11.20303962 | 10.94068065 |
| Chem R23 | 7.179663217 | 10.58248176 | 10.30520676 |
| Chordin-Like 1 | 6.501073196 | 10.0366257 | 11.33831855 |
| Chordin-Like 2 | 8.390166248 | 9.809798285 | 9.847840356 |
| CLC | 7.891019714 | 11.39850062 | 11.27190067 |
| CNTF | 10.04468285 | 13.29806998 | 12.81658371 |
| CNTF R alpha | 9.493799961 | 11.10015228 | 10.8181827 |
| Coagulation Factor III / Tissue Factor | 6.823001291 | 11.83927854 | 11.55710466 |
| CRIM 1 | 7.852545566 | 12.18253964 | 12.22987027 |
| Cripto-1 | 0.0581297 | 12.04693065 | 11.69837911 |
| CRTH-2 | 8.026796493 | 8.73096217 | 8.32305476 |
| Cryptic | 8.628934137 | 11.48652681 | 11.18285986 |
| Csk | 10.09533344 | 11.68676029 | 11.85213933 |
| CTACK / CCL27 | 9.6561822 | 13.15098738 | 13.03797586 |
| CTGF / CCN2 | 7.34584208 | 13.27848909 | 12.75952414 |
| CTLA-4 /CD152 | 9.375542314 | 11.81712933 | 11.54785851 |
| CV-2 / Crossveinless-2 | 9.432082355 | 11.38631329 | 11.33119688 |
| CXCL14 / BRAK | 7.665460013 | 10.94122725 | 9.992584344 |
| CXCL16 | 6.301303683 | 9.822668668 | 10.44734157 |
| CXCR1 / IL-8 RA | 7.982942204 | 11.41983294 | 11.81888171 |
| CXCR2 / IL-8 RB | 6.796221959 | 12.56159509 | 11.8992625 |
| CXCR3 | 6.063754249 | 12.64899077 | 12.18526261 |
| CXCR4 (fusin) | 9.74287832 | 12.35067524 | 12.08969874 |
| CXCR5 /BLR-1 | 7.215476635 | 12.23324867 | 12.17827581 |
| CXCR6 | 6.329592728 | 12.1943113 | 12.20051551 |
| D6 | 8.270017994 | 13.55295395 | 13.77730669 |
| DAN | 5.730555042 | 11.94726954 | 12.14752294 |
| DANCE | 6.909878741 | 11.8377876 | 11.76165511 |
| DcR3 / TNFRSF6B | 6.928494419 | 10.67993532 | 10.13699111 |
| Decorin | 7.086035696 | 13.17236422 | 13.83688866 |
| Dkk-1 | 11.75954447 | 14.50756385 | 14.65643883 |
| Dkk-3 | 7.107978249 | 11.34702652 | 11.2701493 |
| Dkk-4 | 9.341796867 | 11.05835601 | 11.02980485 |
| DR3 / TNFRSF25 | 8.577765953 | 9.862805707 | 9.439830884 |
| DR6 / TNFRSF21 | 6.597288511 | 9.338180281 | 10.52160044 |
| Dtk | 0.0581297 | 12.06520277 | 11.06524782 |
| EDA-A2 | 15.24284042 | 14.61131375 | 14.98392881 |
| EDAR | 5.839489413 | 11.08074542 | 10.35397382 |
| EDG-1 | 9.670537493 | 12.24017819 | 11.93985465 |
| EGF | 8.41458167 | 12.08088506 | 12.14362237 |
| EGF R / ErbB1 | 5.650586737 | 9.954397219 | 10.31004468 |
| EG-VEGF / PK1 | 3.612718551 | 13.13434582 | 13.93182246 |
| EMAP-II | 8.678349525 | 11.90988859 | 11.29963733 |
| ENA-78 | 5.620372124 | 11.18248292 | 11.8622499 |
| Endocan | 9.936180613 | 11.54251451 | 11.58061189 |
| Endoglin / CD105 | 8.789448731 | 11.48545601 | 11.50469487 |
| Endostatin | 8.233055383 | 11.77314959 | 12.42966827 |
| Endothelin | 8.462206836 | 12.54026882 | 12.20181762 |
| EN-RAGE | 7.174473661 | 9.607245531 | 9.918863237 |
| Eotaxin/ CCL11 | 2.813017202 | 10.4829815 | 10.8045344 |
| Eotaxin-2/ MPIF-2 | 0.0581297 | 11.49518297 | 12.13923157 |
| Eotaxin-3/ CCL26 | 0.005919507 | 11.20188108 | 12.26656696 |
| Epiregulin | 6.467520636 | 13.45211398 | 13.38650247 |
| ErbB2 | 0.263528627 | 11.48020951 | 11.19644844 |
| ErbB3 | 0.014942467 | 11.98866935 | 12.68310427 |
| ErbB4 | 5.965020295 | 11.67376175 | 11.92091107 |
| Erythropoietin? | 11.38026318 | 12.34622535 | 12.14162821 |
| E-Selectin | 7.897333488 | 12.39646213 | 12.44138803 |
| FACX | 8.635558528 | 9.418173134 | 10.34040649 |
| FADD | 5.334254105 | 10.90181569 | 10.46887855 |
| FAM3B | 11.41017313 | 11.49423688 | 11.4603279 |
| Fas / TNFRSF6 | 6.862260721 | 9.60637017 | 9.758639637 |
| Fas Ligand | 6.813017202 | 11.94207507 | 12.58836329 |
| FGF Basic | 6.778533343 | 11.66085585 | 11.57978733 |
| FGF R3 | 7.276805772 | 12.43042974 | 12.81838246 |
| FGF R4 | 5.839489413 | 12.93214383 | 12.80327346 |
| FGF R5 | 8.193838986 | 10.78095098 | 10.436191 |
| FGF-10 / KGF-2 | 7.349046584 | 12.2962808 | 11.83427352 |
| FGF-11 | 8.921213413 | 13.67110392 | 13.41031884 |
| FGF-12 | 7.912364143 | 10.90217238 | 11.00140819 |
| FGF-13 1B | 8.633091932 | 11.82005823 | 11.75676479 |
| FGF-16 | 9.319637009 | 11.85879756 | 11.724727 |
| FGF-17? | 8.484803079 | 10.85578313 | 10.87075 |
| FGF-18? | 9.718125592 | 11.15655506 | 11.24421516 |
| FGF-19? | 9.117474161 | 9.517435181 | 10.12250448 |
| FGF-20 | 8.910659209 | 10.64135562 | 10.08381171 |
| FGF-21 | 4.772375218 | 9.149163247 | 8.686500527 |
| FGF-23 | 8.288150136 | 11.80678346 | 12.63469763 |
| FGF-4? | 5.839489413 | 11.02057902 | 11.35093918 |
| FGF-5 | 5.975779826 | 11.72032182 | 11.54182262 |
| FGF-6? | 4.799596686 | 11.74200447 | 11.75133484 |
| FGF-7 / KGF | 7.674678544 | 12.22834969 | 13.09259199 |
| FGF-8? | 6.432284977 | 11.17172389 | 11.21158415 |
| FGF-9? | 5.70198589 | 12.16607449 | 11.61194694 |
| FGF-BP | 7.472001375 | 12.5148956 | 12.80281925 |
| FLRG | 2.865484622 | 10.30181228 | 10.27554256 |
| Flt-3 Ligand? | 8.927723543 | 11.80830677 | 12.04831657 |
| Follistatin | 11.05707265 | 12.97414336 | 14.59633537 |
| Follistatin-like 1 | 11.28589604 | 14.26859414 | 14.07382776 |
| Fractalkine? | 6.140278741 | 12.236364 | 11.8181827 |
| Frizzled-1 | 9.683838543 | 6.857515327 | 6.985841937 |
| Frizzled-3 | 9.2736627 | 8.94550485 | 8.724513853 |
| Frizzled-4 | 10.39464575 | 8.740568285 | 8.581200582 |
| Frizzled-5 | 6.819680932 | 10.0202878 | 10.17492568 |
| Frizzled-6 | 6.097048689 | 9.507154298 | 9.364681856 |
| Frizzled-7 | 7.182251011 | 9.359117891 | 9.124121312 |
| Galectin-3 | 8.669154497 | 10.81241431 | 12.62964121 |
| GASP-1 / WFIKKNRP | 10.59341335 | 12.70388278 | 12.24034415 |
| GASP-2 / WFIKKN | 10.76635877 | 11.36538146 | 9.769837844 |
| GCP-2 / CXCL6 | 5.573829538 | 9.066921253 | 9.203348003 |
| GCSF | 9.353285573 | 10.2511884 | 10.4812949 |
| G-CSF R / CD 114 | 7.375542313 | 11.84596878 | 12.02963233 |
| GDF1 | 10.84969961 | 9.401617511 | 9.01402047 |
| GDF11 | 11.53436961 | 11.38388896 | 11.14688684 |
| GDF-15 | 5.918722222 | 12.07615082 | 12.82828527 |
| GDF3 | 11.97941279 | 11.47218368 | 10.87862573 |
| GDF5 | 11.98849745 | 13.11835461 | 13.41527963 |
| GDF8 | 11.25103892 | 10.92446824 | 10.95128471 |
| GDF9 | 11.83332843 | 9.115104677 | 8.978710459 |
| GDNF? | 7.153526722 | 11.43075289 | 12.01314817 |
| GFR alpha-1 | 5.744630227 | 9.377705404 | 9.355901638 |
| GFR alpha-2 | 7.974009079 | 10.82976859 | 10.63254088 |
| GFR alpha-3 | 9.698826537 | 12.33636061 | 12.00369358 |
| GFR alpha-4 | 11.4206517 | 11.55266095 | 11.20197073 |
| GITR / TNFRF18 | 10.3326905 | 11.50777763 | 11.92546172 |
| GITR Ligand / TNFSF18 | 9.845216024 | 11.39102269 | 11.27873983 |
| GLO-1 | 9.324330612 | 12.3720775 | 13.21621536 |
| Glucagon | 8.55098432 | 10.16285423 | 9.851749041 |
| Glut1 | 9.454734481 | 10.69380604 | 12.02401418 |
| Glut2 | 10.83452791 | 11.29554971 | 10.96920662 |
| Glut3 | 10.53599516 | 10.69504171 | 11.16553514 |
| Glut5 | 10.50779424 | 11.86439164 | 11.76217438 |
| Glypican 3 | 13.61598226 | 14.70581646 | 15.07177658 |
| Glypican 5 | 7.652454304 | 11.15939274 | 11.38046107 |
| GM-CSF | 10.0407547 | 11.19082838 | 10.83130724 |
| GM-CSF R alpha | 8.360071425 | 12.02498899 | 12.70065645 |
| Granzyme A | 11.58902368 | 8.906211572 | 8.812177306 |
| Grb2 | 9.43534023 | 9.508091957 | 8.691743519 |
| GREMLIN | 13.42837437 | 11.59861293 | 11.47548013 |
| GRO | 11.44722322 | 13.00061848 | 13.26631074 |
| GRO-a | 7.846032259 | 13.33618812 | 13.93447411 |
| Growth Hormone (GH) | 6.129592062 | 12.49457828 | 12.452563 |
| Growth Hormone R (GHR) | 9.353898634 | 11.21083547 | 11.37313704 |
| HB-EGF | 7.719545469 | 11.79088343 | 12.35114629 |
| HCC-4?/ CCL16 | 7.60116152 | 10.28959455 | 10.63503787 |
| HCR / CRAM-A/B | 10.48019447 | 12.04886496 | 12.35507575 |
| Hepassocin | 10.89260411 | 12.4110103 | 11.75028827 |
| HGF? | 7.144513279 | 12.73755265 | 12.26260874 |
| HGFR | 11.67352944 | 10.15299672 | 10.47370575 |
| HRG-alpha? | 17.18942903 | 9.255725176 | 9.301496195 |
| HRG-beta?1 | 4.415681704 | 9.750020716 | 9.796039609 |
| HVEM / TNFRSF14 | 7.507278345 | 11.4230679 | 11.5195134 |
| I-309 | 0.005919507 | 9.935005061 | 9.994706995 |
| ICAM-1 | 5.651007255 | 10.83612967 | 11.28048081 |
| ICAM-2 | 2.581691656 | 9.787574404 | 9.346513733 |
| ICAM-3 (CD50) | 5.70198589 | 8.11893213 | 7.836050355 |
| ICAM-5 | 8.314098426 | 11.08043025 | 10.28366717 |
| IFN-alpha / beta R1 | 8.911597868 | 10.56005214 | 10.98013958 |
| IFN-alpha / beta R2 | 7.946872949 | 8.724522374 | 8.707359132 |
| IFN-beta? | 8.272448821 | 11.65515317 | 11.60733031 |
| IFN-gamma | 11.35447238 | 11.78847359 | 11.06827446 |
| IFN-gamma R1 | 8.144535862 | 12.4927439 | 12.67054561 |
| IGFBP-1? | 7.873512995 | 11.86384236 | 12.30648896 |
| IGFBP-2? | 7.492757927 | 11.91104107 | 11.66044165 |
| IGFBP-3? | 8.328425026 | 13.2409459 | 13.59563629 |
| IGFBP-4? | 10.65142983 | 11.79261602 | 12.33985 |
| IGFBP-6 | 7.794531631 | 12.3193674 | 12.04140248 |
| IGFBP-rp1 / IGFBP-7 | 13.90719266 | 15.86070627 | 14.97926888 |
| IGF-I | 6.537909964 | 10.92762542 | 10.56890615 |
| IGF-I SR | 7.729145965 | 10.33177905 | 10.4314976 |
| IGF-II | 8.240524053 | 11.06330839 | 11.6110248 |
| IGF-II R | 9.490149547 | 10.65454222 | 10.36686859 |
| IL-1 alpha | 11.71589474 | 11.21543543 | 10.80009099 |
| IL-1 beta | 7.488582251 | 11.02844272 | 11.23810614 |
| IL-1 F10 / IL-1HY2 | 10.54355824 | 9.181848803 | 8.884170519 |
| IL-1 F5 / FIL1delta | 8.854169309 | 11.79751377 | 12.37524311 |
| IL-1 F6 / FIL1 epsilon | 7.99329475 | 13.41350751 | 13.38414292 |
| IL-1 F7 / FIL1 zeta | 6.669154497 | 12.59176147 | 12.94338477 |
| IL-1 F8 / FIL1 eta | 8.829619169 | 13.11312581 | 13.12157399 |
| IL-1 F9 / IL-1 H1 | 7.130004716 | 12.80314826 | 11.65485975 |
| IL-1 R3 / IL-1 R AcP | 3.327847602 | 10.34862754 | 10.60316268 |
| IL-1 R4 / ST2 | 7.774440578 | 8.683608999 | 7.981567282 |
| IL-1 R6 / IL-1 Rrp2 | 10.32603226 | 9.815111767 | 9.566529783 |
| IL-1 R8 | 8.450447123 | 9.130408829 | 9.029287227 |
| IL-1 R9 | 6.68748632 | 9.761075216 | 9.662668376 |
| IL-1 ra? | 6.994767639 | 11.63887207 | 12.73110685 |
| IL-1 sRI | 9.924635912 | 10.55914766 | 11.04302728 |
| IL-1 sRII | 8.446146985 | 11.10836817 | 11.02410078 |
| IL-10 | 9.306981829 | 10.97705338 | 10.74357219 |
| IL-10 R alpha? | 9.234926178 | 10.58470566 | 10.64588355 |
| IL-10 R beta? | 9.254731826 | 8.705028695 | 8.133142212 |
| IL-11 | 9.169906341 | 12.28326303 | 12.63327907 |
| IL-12 p40 | 6.965020295 | 10.03987119 | 9.495355392 |
| IL-12 p70 | 10.40962111 | 11.23056889 | 11.02928723 |
| IL-12 R beta 1 | 7.844399328 | 12.29397208 | 11.91345044 |
| IL-12 R beta 2 | 11.94725336 | 10.77823303 | 11.5602134 |
| IL-13 | 10.19316489 | 10.62329543 | 10.37937837 |
| IL-13 R alpha 1 | 9.418976784 | 11.47841656 | 11.63526466 |
| IL-13 R alpha 2 | 6.643092201 | 10.50146196 | 10.71059122 |
| IL-15 | 11.27850803 | 11.57240607 | 11.71478213 |
| IL-15 R alpha | 7.274875558 | 12.36251816 | 11.7638348 |
| IL-16? | 7.240524054 | 10.19668922 | 10.30720081 |
| IL-17 | 9.22529265 | 10.99371805 | 10.9436137 |
| IL-17B | 7.471757629 | 13.72187142 | 14.10320583 |
| IL-17B R | 9.438049517 | 12.60639248 | 12.11861669 |
| IL-17C | 7.712765729 | 11.53655306 | 11.68737568 |
| IL-17D | 9.868299638 | 10.50967815 | 10.77272695 |
| IL-17E | 7.665460013 | 13.30776166 | 12.82396426 |
| IL-17F | 7.240524054 | 11.43852012 | 11.60420572 |
| IL-17R | 8.317872964 | 12.49617407 | 11.87305955 |
| IL-17RC | 11.28499342 | 12.07377787 | 12.82520725 |
| IL-17RD | 9.033977668 | 9.112640877 | 9.137631598 |
| IL-18 BPa? | 7.60502416 | 11.79040178 | 12.28648557 |
| IL-18 R alpha /IL-1 R5 | 6.751616658 | 11.84429913 | 12.43768706 |
| IL-18 R beta?/AcPL | 7.659900488 | 11.1185343 | 11.35782688 |
| IL-19 | 8.245481773 | 12.99703292 | 13.55401919 |
| IL-2 | 11.74747255 | 10.84376327 | 10.66111003 |
| IL-2 R alpha? | 8.343531918 | 10.05599004 | 10.59851781 |
| IL-2 R beta?/CD122 | 9.261602146 | 11.20477567 | 11.1411492 |
| IL-2 R gamma | 9.135081147 | 12.1845938 | 11.90801726 |
| IL-20 | 6.819680932 | 12.03136204 | 11.76341987 |
| IL-20 R alpha | 9.445608566 | 11.58468039 | 10.9569206 |
| IL-20 R beta | 8.527771517 | 10.14698952 | 9.908767883 |
| IL-21 | 11.74528664 | 12.04491297 | 11.52845411 |
| IL-21 R? | 6.891019715 | 9.992751629 | 10.32811389 |
| IL-22 | 7.0581297 | 11.44856761 | 11.48230378 |
| IL-22 BP | 7.561955438 | 11.23652153 | 11.40726776 |
| IL-22 R | 8.255596328 | 9.451700151 | 9.194756854 |
| IL-23 | 7.960504814 | 7.539010778 | 7.796039609 |
| IL-23 R | 8.016682415 | 8.91754785 | 9.30035256 |
| IL-24 | 8.355045907 | 11.29473488 | 12.03101191 |
| IL-26 | 8.562948688 | 10.52597148 | 9.822570831 |
| IL-27 | 10.14260594 | 9.255166798 | 9.370142479 |
| IL-28A | 7.670998197 | 8.993162706 | 9.358101707 |
| IL-29 | 7.573829538 | 12.77138235 | 13.39560107 |
| IL-3 | 6.529162809 | 10.91247837 | 10.77437527 |
| IL-3 R alpha | 6.639330282 | 11.25758268 | 10.59712143 |
| IL-31 | 6.878308663 | 7.065445744 | 1 |
| IL-31 RA | 7.400204368 | 7.420506905 | 7.400879436 |
| IL-4 | 6.581691656 | 11.91802483 | 12.15331452 |
| IL-4 R | 5.891019714 | 12.45659029 | 12.67727919 |
| IL-5 | 11.68967289 | 11.67146184 | 11.46709603 |
| IL-5 R alpha? | 8.507278345 | 11.00638179 | 11.13169622 |
| IL-6 | 11.22899365 | 11.56047847 | 11.90284614 |
| IL-6 R | 7.310795132 | 10.01765649 | 9.85408918 |
| IL-7 | 11.56815206 | 11.54114097 | 11.27946549 |
| IL-7 R alpha | 7.021025706 | 9.937095304 | 10.48507456 |
| IL-8 | 11.10729757 | 12.42442024 | 12.45327063 |
| IL-9 | 10.60043612 | 10.54482681 | 10.65172443 |
| Insulin | 5.658042542 | 10.51994113 | 10.61677855 |
| Insulin R | 12.37497688 | 10.71060265 | 10.74378254 |
| Insulysin / IDE | 8.545969734 | 9.700840875 | 10.68650053 |
| IP-10? | 0.0581297 | 8.594600916 | 9.30492167 |
| I-TAC / CXCL11 | 8.590485625 | 9.963654686 | 14.01009097 |
| Kininostatin / kininogen | 8.344687461 | 9.677692625 | 10.18920683 |
| Kremen-1 | 10.1816045 | 9.594941873 | 9.399811959 |
| Kremen-2 | 9.803804024 | 9.39098233 | 9.639340709 |
| Latent TGF-beta bp1? | 8.94915389 | 12.61207277 | 11.27844946 |
| LBP | 9.117474161 | 8.003989577 | 7.936637939 |
| Lck | 10.91455523 | 8.831770192 | 9.15797845 |
| LECT2? | 5.541945477 | 7.373946519 | 5.686500527 |
| Lefty - A | 7.105253612 | 10.47604973 | 10.52135506 |
| Leptin (OB) | 7.53183545 | 8.575047417 | 8.033423002 |
| Leptin R | 8.549982796 | 9.639266087 | 10.26238852 |
| LFA-1 alpha? | 8.917664486 | 8.026414093 | 7.836050355 |
| LIF R alpha | 7.646081927 | 9.808276546 | 10.17023805 |
| LIF? | 4.56592434 | 9.839900774 | 9.932952892 |
| LIGHT / TNFSF14 | 8.039696982 | 10.89089389 | 11.40127954 |
| Lipocalin-1 | 8.847663345 | 9.820405751 | 10.01785233 |
| Lipocalin-2 | 9.364761062 | 11.01992177 | 13.21352196 |
| LRP-1 | 10.60598821 | 9.267955118 | 9.89216331 |
| LRP-6 | 11.64742973 | 7.991975142 | 8.071462363 |
| L-Selectin (CD62L) | 10.32169234 | 10.98364125 | 11.18006454 |
| Lymphotactin?/ XCL1 | 7.696565614 | 11.75943401 | 10.56176526 |
| Lymphotoxin beta / TNFSF3 | 7.775806123 | 11.68250966 | 11.89671082 |
| Lymphotoxin beta R / TNFRSF3 | 7.90675264 | 11.31067608 | 10.49535539 |
| MAC-1? | 10.08188405 | 8.350898159 | 8.935901682 |
| MCP-1 | 11.27517861 | 11.90357802 | 13.01820018 |
| MCP-2 | 9.251038919 | 8.748524809 | 9.861474675 |
| MCP-3 | 8.521654073 | 12.44028794 | 11.88445674 |
| MCP-4 / CCL13 | 8.821342067 | 3.969565521 | 5.199672345 |
| M-CSF | 7.343531919 | 10.53059311 | 11.19121347 |
| M-CSF R? | 8.808836686 | 10.05631059 | 10.25561875 |
| MDC | 6.779228888 | 8.236746719 | 9.180530807 |
| MFG-E8 | 8.257802045 | 10.85651955 | 10.61562964 |
| MFRP | 9.824244457 | 9.260184432 | 9.271463028 |
| MICA | 8.350451332 | 9.946464101 | 9.700439718 |
| MIF? | 8.529804914 | 10.12851154 | 11.11211366 |
| MIG | 11.18063418 | 10.46087532 | 10.37585398 |
| MIP 2 | 11.58719417 | 13.77974814 | 13.96135925 |
| MIP-1a | 8.447223222 | 9.312110559 | 10.64295422 |
| MIP-1b | 10.94858399 | 11.89625523 | 12.14362237 |
| MIP-1d | 8.086035696 | 8.190768848 | 7.857980995 |
| MIP-3 alpha? | 8.26514402 | 7.743937232 | 8.674192268 |
| MIP-3 beta? | 11.58999847 | 6.050662371 | 6.991521846 |
| MMP-1? | 11.32798689 | 11.10759508 | 13.36720996 |
| MMP-10? | 9.752487587 | 11.00803983 | 11.603974 |
| MMP-11 /Stromelysin-3 | 9.430450655 | 12.14641133 | 13.34474668 |
| MMP-12? | 8.960658124 | 9.575840273 | 10.17741954 |
| MMP-13? | 8.503144546 | 9.167676991 | 8.666224003 |
| MMP-14? | 9.046104224 | 11.6985137 | 12.00281502 |
| MMP-15 | 8.160105371 | 12.04693065 | 12.58871464 |
| MMP-16 / MT3-MMP | 9.495361601 | 11.13121812 | 11.70108982 |
| MMP-19 | 10.60526523 | 9.631542465 | 10.4236412 |
| MMP-2? | 9.924635912 | 11.19214168 | 11.88502901 |
| MMP-20 | 13.14642106 | 12.14618548 | 12.4105176 |
| MMP-24 / MT5-MMP | 9.531835449 | 5.767123958 | 6.689997971 |
| MMP-25 / MT6-MMP | 8.095676654 | 11.1613311 | 9.884552134 |
| MMP-3 | 10.82796754 | 8.025104609 | 11.97486267 |
| MMP-7? | 7.922315845 | 11.07237024 | 11.85301709 |
| MMP-8? | 11.16779714 | 8.733369702 | 10.06709819 |
| MMP-9? | 8.952189546 | 9.987718647 | 10.18239435 |
| MSP alpha Chain | 9.781790644 | 6.715362606 | 7.958552715 |
| Musk | 11.7855184 | 12.13795561 | 12.69228857 |
| NAP-2? | 8.225547846 | 9.834677975 | 10.74041323 |
| NCAM-1 / CD56 | 9.825486554 | 12.16436555 | 12.28576343 |
| Neuritin? | 8.294610107 | 11.06903828 | 11.32502431 |
| NeuroD1 | 7.441833993 | 8.731765127 | 9.448116305 |
| Neuropilin-2 | 7.669154497 | 10.23485549 | 10.47927503 |
| Neurturin? | 8.03254429 | 11.33940795 | 12.10451685 |
| NGF R? | 9.386243595 | 10.11872269 | 10.58120058 |
| Nidgen-1 | 8.871910891 | 10.79331112 | 11.30862345 |
| NOV / CCN3 | 10.20342504 | 10.0431094 | 12.94347635 |
| NrCAM | 9.252886554 | 12.00087777 | 12.46377979 |
| NRG1 Isoform GGF2 | 8.579730139 | 6.617965886 | 7.525520809 |
| NRG2 | 11.82092696 | 9.428115158 | 9.158609688 |
| NRG3 | 12.18338171 | 11.23042686 | 11.40287883 |
| NT-3? | 8.559966885 | 7.201506092 | 7.73978061 |
| NT-4? | 8.285745643 | 9.258513826 | 9.695228291 |
| Orexin A? | 7.691124897 | 7.156766131 | 8.476746204 |
| Orexin B | 9.943826073 | 12.54370228 | 12.57323297 |
| OSM | 8.007362862 | 10.54984391 | 11.37082361 |
| Osteoactivin / GPNMB | 8.268801044 | 8.846672448 | 9.364681856 |
| Osteocrin | 9.536899319 | 9.019047558 | 8.997179481 |
| Osteoprotegerin / TNFRSF11B | 10.23130924 | 15.45299161 | 15.69892166 |
| OX40 Ligand / TNFSF4 | 8.56592434 | 12.64989375 | 11.87392467 |
| PARC / CCL18 | 10.75357551 | 9.971494092 | 10.15734694 |
| PD-ECGF | 8.798753744 | 11.4935269 | 11.19629475 |
| PDGF R alpha? | 11.09653433 | 10.6252413 | 10.60130653 |
| PDGF R beta? | 10.66360922 | 11.36019807 | 11.3027817 |
| PDGF-AA | 8.886266184 | 9.150364879 | 9.281350515 |
| PDGF-AB | 8.592432582 | 9.843619803 | 9.732591436 |
| PDGF-BB | 7.965020295 | 7.948428121 | 7.670656249 |
| PDGF-C | 8.786050154 | 8.301996757 | 8.764042217 |
| PDGF-D | 7.488582251 | 6.712111296 | 7.769837844 |
| PECAM-1 /CD31 | 8.289350881 | 7.382152677 | 8.465566405 |
| Pentraxin3 / TSG-14 | 8.857411321 | 11.39089561 | 14.25369978 |
| Persephin | 9.347572276 | 9.312110559 | 9.556506055 |
| PF4?/ CXCL4 | 10.28815014 | 12.5961774 | 13.00140819 |
| PlGF? | 10.50133228 | 8.086656489 | 9.682555741 |
| PLUNC | 9.219891443 | 10.52179931 | 10.8372337 |
| Prdx6 | 7.949913403 | 10.23796736 | 10.01959073 |
| Pref-1 | 8.608876485 | 12.03429398 | 12.57080444 |
| Progranulin | 12.5605886 | 11.58767728 | 15.20935817 |
| Prolactin? | 9.777518521 | 11.98109181 | 12.20670915 |
| P-selectin | 10.9853521 | 8.318125704 | 8.298062568 |
| RAGE | 8.554983477 | 10.34443383 | 10.76051205 |
| RANK / TNFRSF11A | 9.246100292 | 9.891473833 | 9.678600139 |
| RANTES | 7.698374636 | 8.990481399 | 9.291170699 |
| RELM beta | 9.357337718 | 8.733369702 | 9.056637715 |
| RELT / TNFRSF19L | 10.82258666 | 11.2911986 | 11.760616 |
| ROBO4 | 8.689306756 | 6.296235356 | 7.2644426 |
| S100 A8/A9 | 11.82765765 | 11.28273079 | 11.64205169 |
| S100A10 | 7.233055383 | 7.203822828 | 8.174925683 |
| SAA | 10.62632335 | 10.79964318 | 11.66099865 |
| SCF | 8.15747751 | 11.89410493 | 12.69484755 |
| SCF R?/CD117 | 8.94915389 | 10.69442401 | 11.43136701 |
| SDF-1 / CXCL12 | 10.62656089 | 11.56971411 | 11.29462075 |
| sFRP-1 | 6.946872949 | 7.833437541 | 7.339850003 |
| sFRP-3 | 6.70198589 | 9.601106818 | 10.41204051 |
| sFRP-4 | 12.67559717 | 12.7808694 | 12.983439 |
| sgp130 | 11.5988673 | 11.31134768 | 11.86882255 |
| SIGIRR | 8.353898634 | 12.35126317 | 12.57246339 |
| Siglec-5/CD170 | 10.22680182 | 9.956459574 | 10.06608919 |
| Siglec-9 | 10.8586252 | 12.3322634 | 12.93401331 |
| SLPI? | 10.42636331 | 10.11257076 | 9.995413852 |
| Smad 1 | 7.424451914 | 8.065155938 | 7.537218401 |
| Smad 4 | 8.026796493 | 9.245078872 | 9.689124405 |
| Smad 5 | 7.310795132 | 8.566971242 | 11.46288565 |
| Smad 7 | 9.137614484 | 9.92521035 | 10.91064273 |
| Smad 8 | 7.368742481 | 7.548156522 | 8.211888295 |
| Soggy-1 | 9.576782856 | 10.86898158 | 10.89102419 |
| Sonic Hedgehog (Shh N-terminal) | 9.926566456 | 8.867280336 | 8.933690655 |
| SPARC? | 14.3350901 | 14.25011324 | 14.93312584 |
| Spinesin | 10.75292285 | 8.896218728 | 9.482807957 |
| TACI / TNFRSF13B | 10.61175899 | 11.02844272 | 11.03514275 |
| Tarc | 9.719907798 | 5.543924475 | 7.011227255 |
| TCCR / WSX-1 | 9.013052992 | 7.945662291 | 7.792790294 |
| TECK?/ CCL25 | 9.34237545 | 9.249010341 | 9.417325118 |
| TFPI | 9.823001291 | 7.800139402 | 7.074141463 |
| TGF-alpha | 8.976247551 | 8.092919678 | 7.74819285 |
| TGF-beta 1 | 11.4323495 | 10.10143075 | 10.2708793 |
| TGF-beta 2? | 9.046236614 | 8.310621296 | 7.957102042 |
| TGF-beta 3? | 8.518377313 | 10.27314162 | 8.381542951 |
| TGF-beta 5 | 11.08965861 | 14.69407113 | 15.54214013 |
| TGF-beta RI / ALK-5 | 8.674678544 | 6.593437261 | 5.714245518 |
| TGF-beta RII | 9.56592434 | 7.845357907 | 8.776433032 |
| TGF-beta RIII | 8.870307005 | 8.957910893 | 9.595257481 |
| Thrombopoietin (TPO) | 9.794531631 | 8.691885022 | 9.368506462 |
| Thrombospondin-1 | 15.63519704 | 17.39880412 | 18.07256051 |
| Thrombospondin-2 | 11.13160185 | 7.231337306 | 7.285402219 |
| Thrombospondin-4 | 9.568399371 | 4.704547735 | 6.044394119 |
| Thymopoietin? | 10.35217601 | 9.193553224 | 9.466586338 |
| Tie-1 | 10.07737292 | 11.25618813 | 11.01854794 |
| Tie-2 | 9.069356955 | 9.180084921 | 9.440869168 |
| TIMP-1? | 11.14459769 | 14.08496213 | 14.58953412 |
| TIMP-2? | 10.54546731 | 5.151212596 | 10.89935692 |
| TIMP-3 | 10.4943207 | 10.96822249 | 11.17305246 |
| TIMP-4 | 8.974009079 | 8.315985568 | 7.912889336 |
| TL1A / TNFSF15 | 8.747254105 | 8.139651626 | 8.52160044 |
| TLR1 | 10.07424154 | 6.721843326 | 7.21916852 |
| TLR2 | 8.620372124 | 3.250316684 | 6.199672345 |
| TLR3 | 9.928494419 | 6.875065601 | 7.985841937 |
| TLR4 | 8.658042542 | 8.430207892 | 9.30035256 |
| TMEFF1 / Tomoregulin-1 | 10.74090488 | 5.580042953 | 2.087462841 |
| TMEFF2 | 11.19959826 | 14.9448522 | 15.60826204 |
| TNF RI / TNFRSF1A | 9.765488832 | 5.852289459 | 1.584962501 |
| TNF RII / TNFRSF1B | 8.397979703 | 12.38374673 | 12.85953479 |
| TNF-alpha | 11.36034021 | 10.75748637 | 10.59828517 |
| TNF-beta | 11.43601803 | 10.60193543 | 10.20548782 |
| TPX | 9.592432582 | 10.37610715 | 10.24108938 |
| TRADD | 10.03612507 | 10.56569232 | 11.09539702 |
| TRAIL R1 / DR4 / TNFRSF10A | 10.94744352 | 10.60193543 | 10.87805091 |
| TRAIL R2?/ DR5 / TNFRSF10B | 10.98516687 | 9.649500345 | 9.385323176 |
| TRAIL R3 / TNFRSF10C | 9.712765728 | 8.696827942 | 8.915879379 |
| TRAIL R4 / TNFRSF10D | 9.314928086 | 7.346949483 | 5.700439718 |
| TRAIL?/ TNFSF10 | 8.523696105 | 9.361195036 | 10.06608919 |
| TRANCE? | 9.460075824 | 10.39928766 | 10.8135808 |
| TREM-1 | 10.14824212 | 8.910473122 | 8.93737382 |
| TROY / TNFRSF19 | 9.359625895 | 8.66523289 | 8.943979914 |
| TSG-6 | 8.857411321 | 7.816884536 | 7.736401931 |
| TSLP R | 8.502109242 | 2.739235401 | 1 |
| TWEAK / TNFSF12 | 10.22492045 | 11.67668362 | 12.32523517 |
| TWEAK R / TNFRSF12 | 9.57874838 | 8.685268006 | 9.692179576 |
| Ubiquitin+1 | 11.56195544 | 12.0088493 | 11.97064518 |
| uPA | 9.556776093 | 9.33923441 | 10.00982862 |
| uPAR | 9.387365442 | 9.728459111 | 9.514714054 |
| Vasorin | 10.49275793 | 8.769790784 | 8.159871337 |
| VCAM-1 (CD106) | 9.875113323 | 9.171824836 | 9.1382718 |
| VE-Cadherin | 10.12489163 | 9.287746785 | 8.983706193 |
| VEGF | 9.507794238 | 9.979291129 | 12.61470984 |
| VEGF R2?(KDR) | 9.276692936 | 8.170775648 | 7.071462363 |
| VEGF R3? | 8.996239026 | 8.264756147 | 7.972979786 |
| VEGF-B | 8.243005043 | 0.960655485 | 2.087462841 |
| VEGF-C? | 9.89812077 | 10.1478922 | 11.66088727 |
| VEGF-D? | 9.681096645 | 5.852289459 | 6.741466986 |
| VEGI?/ TNFSF15 | 9.854169309 | 5.716101137 | 3.129283017 |
| WIF-1 | 9.583650509 | 0.960655485 | 5.781359714 |
| WISP-1 / CCN4 | 10.55747733 | 6.404939964 | 7.164906927 |
| XEDAR | 10.69792259 | 10.65030163 | 8.409390936 |

Table S2: A table of normalized fluorescence signal intensity of neurotrophic factors, growth factors in the CMs of hEF, ASCs and CBMSCs. hEF represents the CM of hEF, ASC represent the CM of ASCs, CBMSC represent the CM of CBMSCs.

| proteinID | HEF | ASC | CBMSC |
| --- | --- | --- | --- |
| BDNF | 2.643092201 | 13.45533821 | 13.52930853 |
| beta-NGF | 2.380057795 | 12.892435 | 13.21219237 |
| CNTF | 10.04468285 | 13.29806998 | 12.81658371 |
| EGF | 8.41458167 | 12.08088506 | 12.14362237 |
| EG-VEGF / PK1 | 3.612718551 | 13.13434582 | 13.93182246 |
| GDNF | 7.153526722 | 11.43075289 | 12.01314817 |
| HGF | 7.144513279 | 12.73755265 | 12.26260874 |
| IGF-I | 6.537909964 | 10.92762542 | 10.56890615 |
| IGF-II | 8.240524053 | 11.06330839 | 11.6110248 |
| Neuritin | 8.294610107 | 11.06903828 | 11.32502431 |
| NeuroD1 | 7.441833993 | 8.731765127 | 9.448116305 |
| Neuropilin-2 | 7.669154497 | 10.23485549 | 10.47927503 |
| Neurturin | 8.03254429 | 11.33940795 | 12.10451685 |
| Nidgen-1 | 8.871910891 | 10.79331112 | 11.30862345 |
| NOV / CCN3 | 10.20342504 | 10.0431094 | 12.94347635 |
| NrCAM | 9.252886554 | 12.00087777 | 12.46377979 |
| NRG1 Isoform GGF2 | 8.579730139 | 6.617965886 | 7.525520809 |
| NRG2 | 11.82092696 | 9.428115158 | 9.158609688 |
| NRG3 | 12.18338171 | 11.23042686 | 11.40287883 |
| NT-3 | 8.559966885 | 7.201506092 | 7.73978061 |
| NT-4 | 8.285745643 | 9.258513826 | 9.695228291 |
| OSM | 8.007362862 | 10.54984391 | 11.37082361 |
| PD-ECGF | 8.798753744 | 11.4935269 | 11.19629475 |
| PDGF-AA | 8.886266184 | 9.150364879 | 9.281350515 |
| PDGF-AB | 8.592432582 | 9.843619803 | 9.732591436 |
| PDGF-BB | 7.965020295 | 7.948428121 | 7.670656249 |
| PDGF-C | 8.786050154 | 8.301996757 | 8.764042217 |
| PDGF-D | 7.488582251 | 6.712111296 | 7.769837844 |
| SCF | 8.15747751 | 11.89410493 | 12.69484755 |
| VEGF | 9.507794238 | 9.979291129 | 12.61470984 |
| VEGF-B | 8.243005043 | 0.960655485 | 2.087462841 |
| VEGF-C | 9.89812077 | 10.1478922 | 11.66088727 |
| VEGF-D | 9.681096645 | 5.852289459 | 6.741466986 |

Table S3: A table of normalized fluorescence signal intensity of the matrix metalloproteinases in the CMs of hEF, ASCs and CBMSCs. hEF represents the CM of hEF, ASC represent the CM of ASCs, CBMSC represent the CM of CBMSCs.

| proteinID | HEF | ASC | CBMSC |
| --- | --- | --- | --- |
| MMP-1 | 11.32798689 | 11.10759508 | 13.36720996 |
| MMP-10 | 9.752487587 | 11.00803983 | 11.603974 |
| MMP-11 / Stromelysin-3 | 9.430450655 | 12.14641133 | 13.34474668 |
| MMP-12 | 8.960658124 | 9.575840273 | 10.17741954 |
| MMP-13 | 8.503144546 | 9.167676991 | 8.666224003 |
| MMP-14 | 9.046104224 | 11.6985137 | 12.00281502 |
| MMP-15 | 8.160105371 | 12.04693065 | 12.58871464 |
| MMP-16 / MT3-MMP | 9.495361601 | 11.13121812 | 11.70108982 |
| MMP-19 | 10.60526523 | 9.631542465 | 10.4236412 |
| MMP-2 | 9.924635912 | 11.19214168 | 11.88502901 |
| MMP-20 | 13.14642106 | 12.14618548 | 12.4105176 |
| MMP-24 / MT5-MMP | 9.531835449 | 5.767123958 | 6.689997971 |
| MMP-25 / MT6-MMP | 8.095676654 | 11.1613311 | 9.884552134 |
| MMP-3 | 10.82796754 | 8.025104609 | 11.97486267 |
| MMP-7 | 7.922315845 | 11.07237024 | 11.85301709 |
| MMP-8 | 11.16779714 | 8.733369702 | 10.06709819 |
| MMP-9 | 8.952189546 | 9.987718647 | 10.18239435 |
